# Longitudinal dynamics of circulating miRNAs in a swine model of familial hypercholesterolemia during early atherosclerosis

**DOI:** 10.1038/s41598-023-46762-0

**Published:** 2023-11-07

**Authors:** Hadjer Namous, Christian Krueger, Yanping Cheng, Pedro H. C. Melo, Athanasios Peppas, Grzegorz L. Kaluza, William C. Stoffregen, Jess Reed, Hasan Khatib, Juan F. Granada

**Affiliations:** 1 Department of Animal and Dairy Sciences, University of Wisconsin Madison, 1675 Observatory Drive, Madison, WI 53706 USA; 2 Skirball Center for Innovation, Cardiovascular Research Foundation, 1700 Broadway, 9th Floor, New York, NY 10019 USA; 3 Northstar Preclinical and Pathology Services, LLC, Lake Elmo, MN USA

**Keywords:** Non-coding RNAs, Transcriptomics, Genetics, Gene expression

## Abstract

Atherosclerosis is a complex progressive disease involving intertwined biological mechanisms. We aimed to identify miRNA expression dynamics at the early stages of atherosclerosis using a large swine model (Wisconsin Miniature Swine, WMS). A total of 18 female pigs; 9 familial hypercholesterolemic (WMS-FH) and 9 normal control swine (WMS-N) were studied. miRNA sequencing was performed on plasma cell-free RNA at 3, 6, and 9 months of age. RT-qPCR validated DE miRNAs in a new cohort of animals (n = 30) with both sexes. Gene ontology and mRNA targets for DE miRNAs were identified. In vivo multimodality imaging and histopathology were performed to document the presence of atherosclerosis at termination. 20, 19, and 9 miRNAs were significantly DE between the groups at months 3, 6, and 9, respectively. Most DE miRNAs and their target genes are involved in human atherosclerosis development. Coronary atherosclerosis was documented in 7/9 WMS-FH pigs. Control animals had no lesions. miR-138, miR-152, miR-190a, and miR-196a showed a significant diagnostic power at month 3, whereas miR-486, miR-126-3p, miR-335, and miR-423-5p were of significant diagnostic power at month 9. In conclusion, specific DE miRNAs with significant discriminatory power may be promising biomarkers for the early detection of coronary atherosclerosis.

## Introduction

Atherosclerosis is a chronic condition that slowly develops over decades. At the early stages of development, disease detection is particularly important to delay the progression of disease. Lipid biomarkers (i.e., LDL-cholesterol) have historically been the standard of care to screen high-risk patients. However, these biomarkers have insufficient predictive value especially amongst individuals with normal cholesterol levels^1^. Other biomarkers such as serum C-reactive protein (CRP), interleukin-6 (IL-6), and tumor necrosis factor-alpha (TNFα) have shown promising results in predicting future cardiac events^2–4^. However, they have not been widely adopted due to their high biological variability in specific patient populations and clinical subsets^2^.

**Figure 1:**
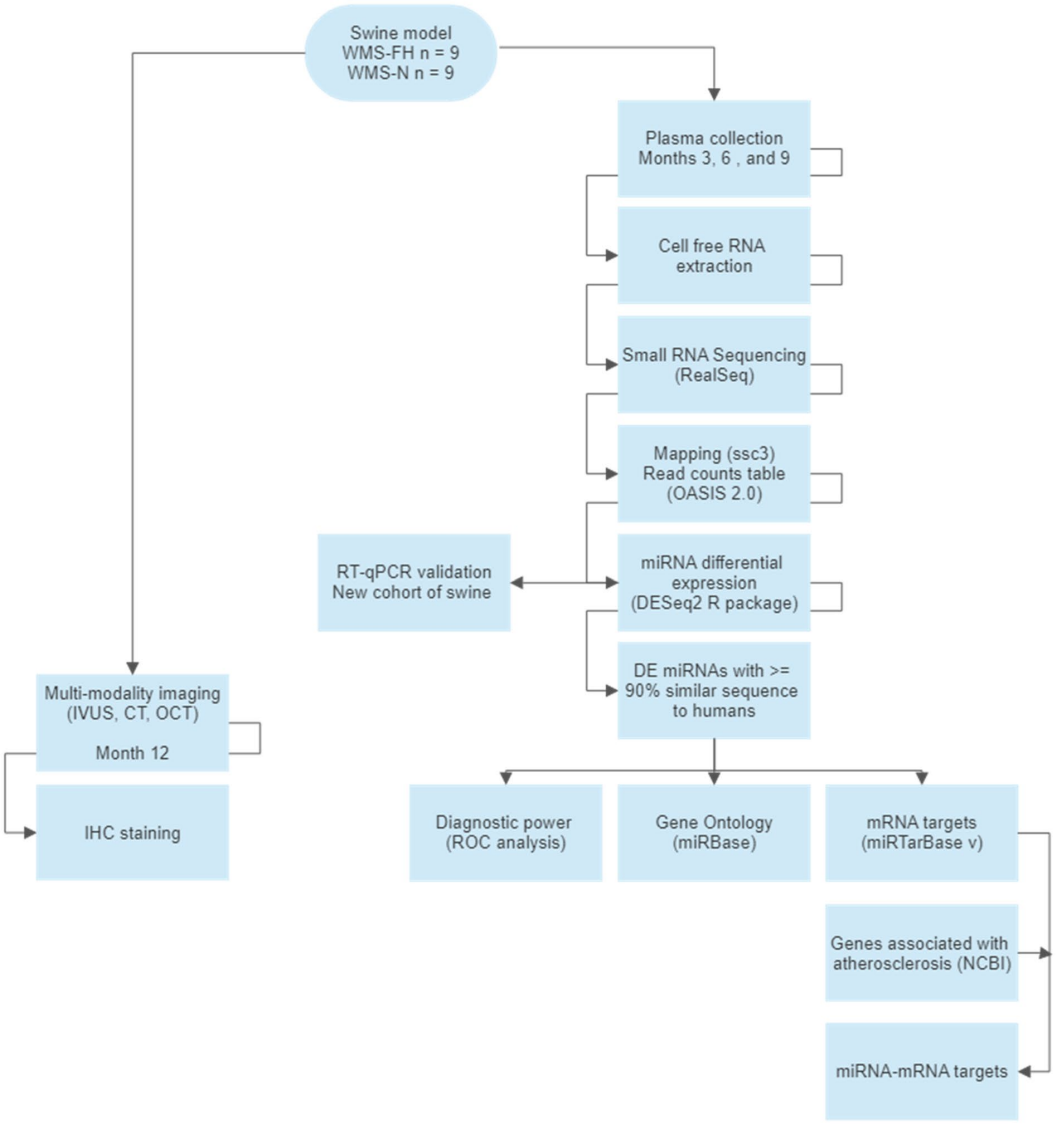
Flow diagram outlining the steps and methods used in the present study.

MicroRNAs (miRNAs) play a role in cardiovascular disease development^5–8^ and are well-suited to be used as biomarkers for atherosclerosis detection. Human studies using circulating miRNAs in atherosclerosis have primarily included patients in which the disease is already present, in an advanced stage or become symptomatic (i.e., acute myocardial infarction)^5,9,10^. Longitudinal studies aimed to characterize the dynamics of circulating miRNA changes at the early stages of disease development are limited. In the present work, we used a well-established model of atherosclerosis (Wisconsin Miniature Swine™ of Familial hypercholesterolemia = WMS-FH)^11–15^ to study; (a) the temporal dynamics of miRNA changes, (b) the miRNA signatures specific to early atherosclerosis development and (c) the diagnostic power of these biomarkers for the detection of the disease. Plasma miRNA levels were assessed at three different time points (3, 6, and 9 months of age) and differentially expressed (DE) miRNAs were compared between WMS-FH and genetically related WMS-N controls. The presence or absence of disease was confirmed by multi-modality imaging and histo-pathology evaluation at 12 months. The diagnostic power of each miRNA in discriminating between the presence and absence of atherosclerosis was calculated and gene ontology (GO) and mRNA targets of DE miRNAs were identified to understand the potential biological mechanisms involved in atherosclerosis development at this early stage. The methodology followed in this work is shown in (Fig. 1).

## Results

### Cholesterol levels in both experimental groups

**Figure 2:**
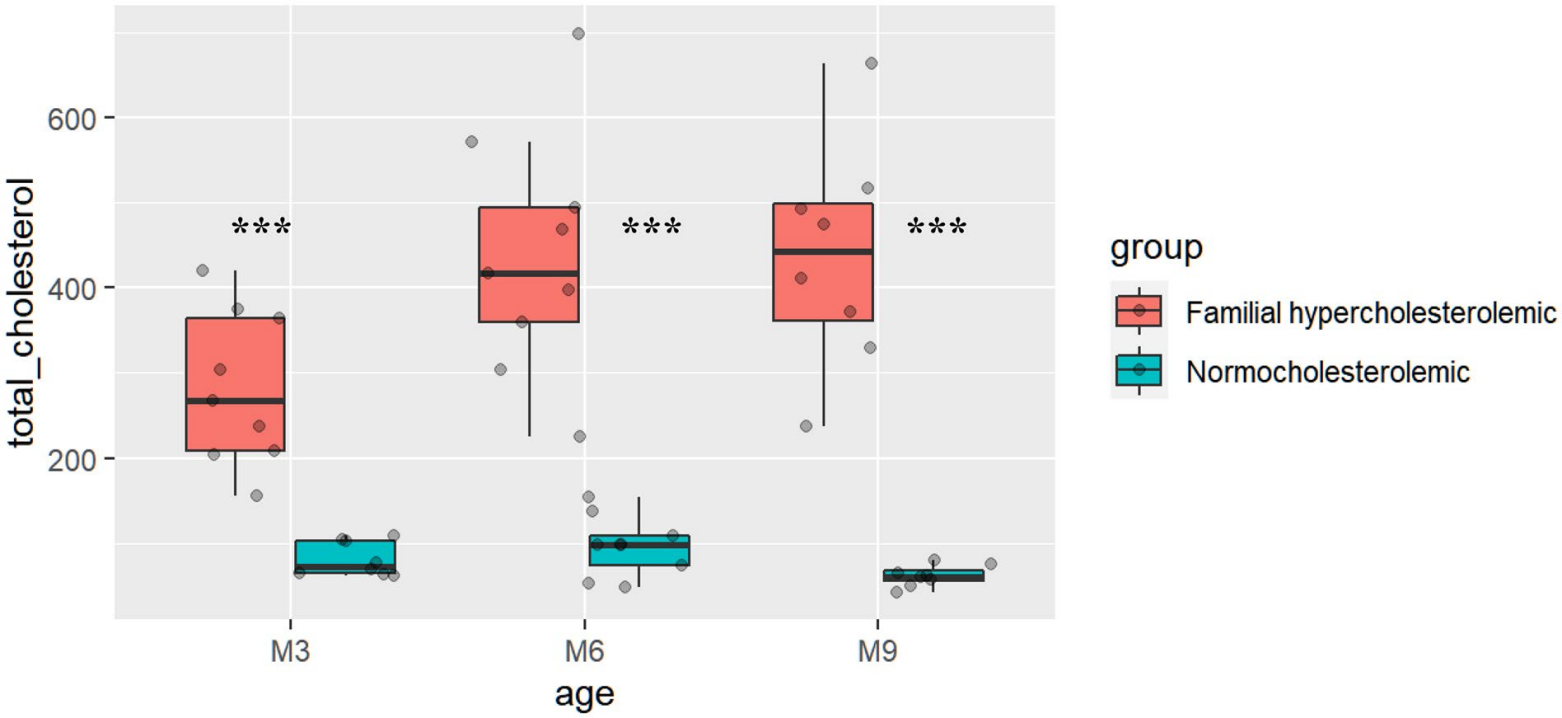
Estimated total cholesterol mg/dL measured using the ELISA method. The boxplots represent total cholesterol levels per group (WMS-FH vs. WMS normal) at each time point (months 3, 6, and 9). The data are for animals recruited in cohort 1. ***p < 0.001.

The characteristics of the animals used for small RNA sequencing are summarized in supplemental Table 2. No significant differences were observed between WMS-FH and WMS-N controls in the weight category. Total cholesterol levels, across the three-time points, were significantly higher in WMS-FH compared to WMS-N (*p* < 0.001). A certain level of variability in total cholesterol levels is commonly seen in WMS-FH model (Fig. 2). Total cholesterol levels in WMS-FH increased from 281.75 ± 89.86 mg/dL at month 3 to 436.9 ± 142.95 mg/dL at month 6 (*p* < 0.05). Cholesterol levels stabilized at 6 months and did not change much by 9-months (month 6 vs. month 9, *p* > 0.1). There were no significant changes in cholesterol levels in the WMS-N control group.

### Multimodality imaging evaluation of atherosclerotic lesions

**Figure 3:**
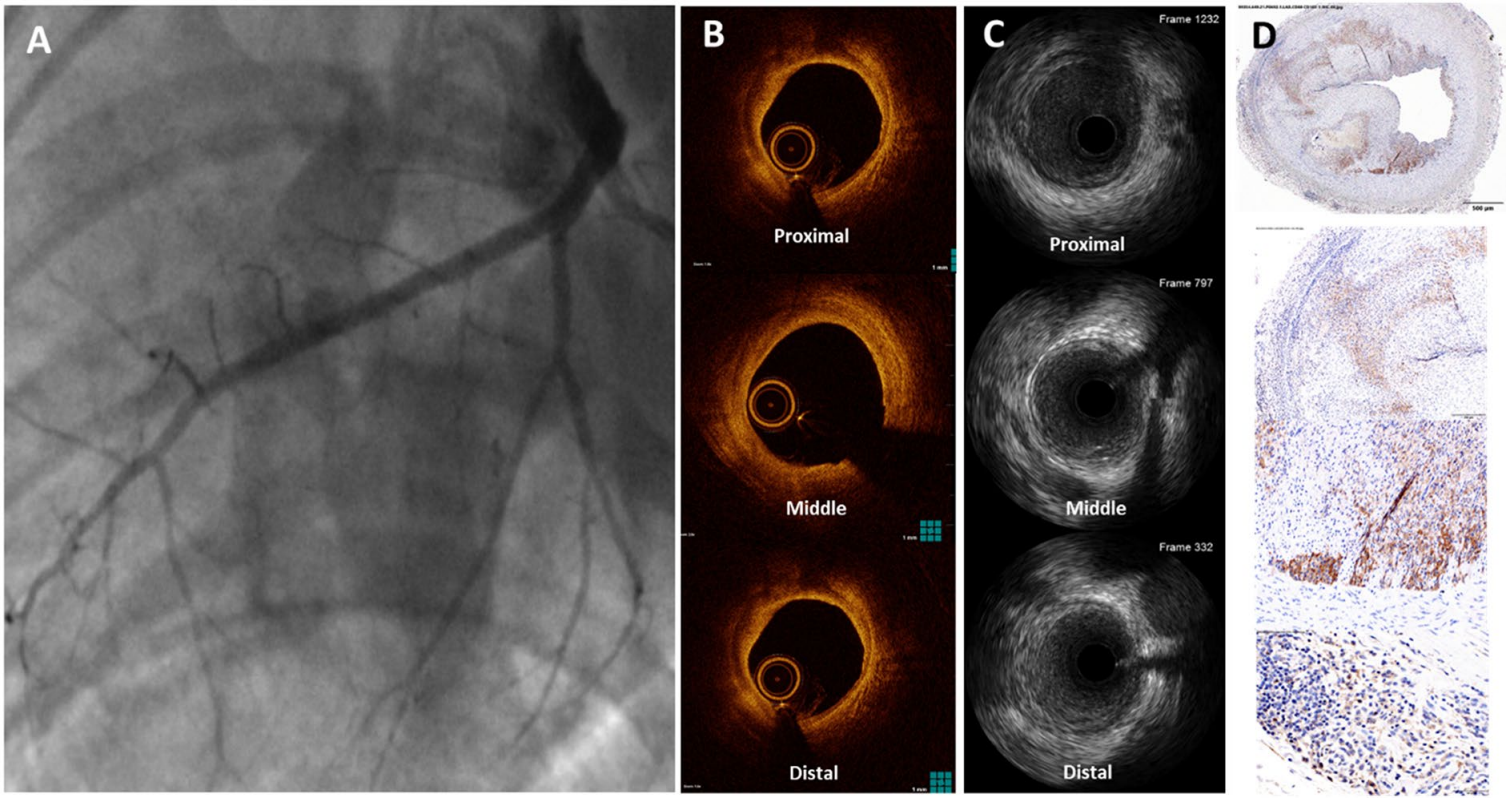
Representative images of angiography (**A**), IVUS (**B**), OCT (**C**), and correlated histological sections (**D**) from a diseased coronary artery.

All animals underwent multimodality imaging with angiography (Fig. 3A), IVUS (Fig. 3B), and OCT (Fig. 3C) at termination of the study (~ 12-months). Evidence of coronary atherosclerosis was documented in 7/9 WMS-FH animals (78%) and in none of the control group animals. No evidence of peripheral atherosclerosis was documented in any of the animals. In the animals with coronary atherosclerosis, the disease was more frequently seen in the right (6/26, 23%) and left anterior descending (5/26, 19%) arteries. Imaging OCT and IVUS analysis results are shown in Table 1. The mean lesion length by OCT was 27 ± 16 mm and the mean percentage area of stenosis was 23% ± 8%.

**Table 1:** Multi-modality imaging analysis.

Optical Coherence Tomography Analysis (n = 11 vessels)	Lesion length (mm)	27.2 ± 16.1
	Lumen area (mm^2^)	9.38 ± 2.52
	Lumen diameter (mm)	3.41 ± 0.50
	Vessel area (mm^2^)	12.07 ± 2.79
	Vessel diameter (mm)	3.88 ± 0.48
	Intimal thickness (mm)	0.24 ± 0.08
	Area stenosis (%)	23 ± 8
Intravascular Ultrasound Analysis (n = 8 vessels)	Minimal lumen diameter (mm)	2.75 ± 0.62
	Minimal lumen area (mm^2^)	7.43 ± 3.53
	Lumen volume (mm^3^)	465.04 ± 400.98
	Vessel volume (mm^3^)	566.16 ± 461.12
	Plaque volume (mm^3^)	98.70 ± 88.22
	Plaque burden (%)	23 ± 9
Quantitative Coronary Angiography (n = 7 vessels)	Minimal lumen diameter (mm)	2.40 ± 0.37
	Reference vessel diameter (mm)	2.92 ± 0.57
	Diameter stenosis (%)	17.45 ± 5.44

### Histopathology evaluation of coronary lesions

A summary of the immunohistochemistry findings by animal is presented in Table 2. Low numbers of evenly dispersed cells within the adventitia and perivascular adipose tissue were positive for CD61/CD163-1 in both study groups regardless the presence of atherosclerosis findings. These cells were slightly more prevalent in perivascular areas and were consistent with resident macrophages.

**Table 2:** Summary of immunohistochemistry results by animal.

Animal	Arteries Examined	Histology Consistent with Atherosclerosis	Arteries Exhibiting Atherosclerotic Changes	CD68/CD163-1 IHC Positive staining						
				% Stained Area	Atherosclerotic IH	Media associated with atherosclerosis	Hypercellular adventitia in levels with atherosclerosis	Nonatherosclerotic IH	Altered media not associated with atherosclerosis	Native Adventitia/Perivascular adipose tissue
WMS-N	LAD, RCA	−	N/A	< 5%	−	−	−	+	+	+
WMS-FH1	LAD, RCA	+	LAD, RCA	10–20%	+	+	+	−	−	+
WMS-FH2	LAD, RCA	+	LAD, RCA	10–20%	+	+	+	−	−	+
WMS-FH3	LAD, RCA	+	LAD	5–10%	+	−	−	−	−	+
WMS-FH4	LAD	+	LAD	5–10%	+	+	−	−	−	+

Sections were stained with anti-CD68 and anti-CD163-1 which were markers for M1 and M2 macrophages.

IH, intimal hyperplasia; LAD, left anterior descending artery; NI, neointima; RCA, right coronary artery.

**Figure 4:**
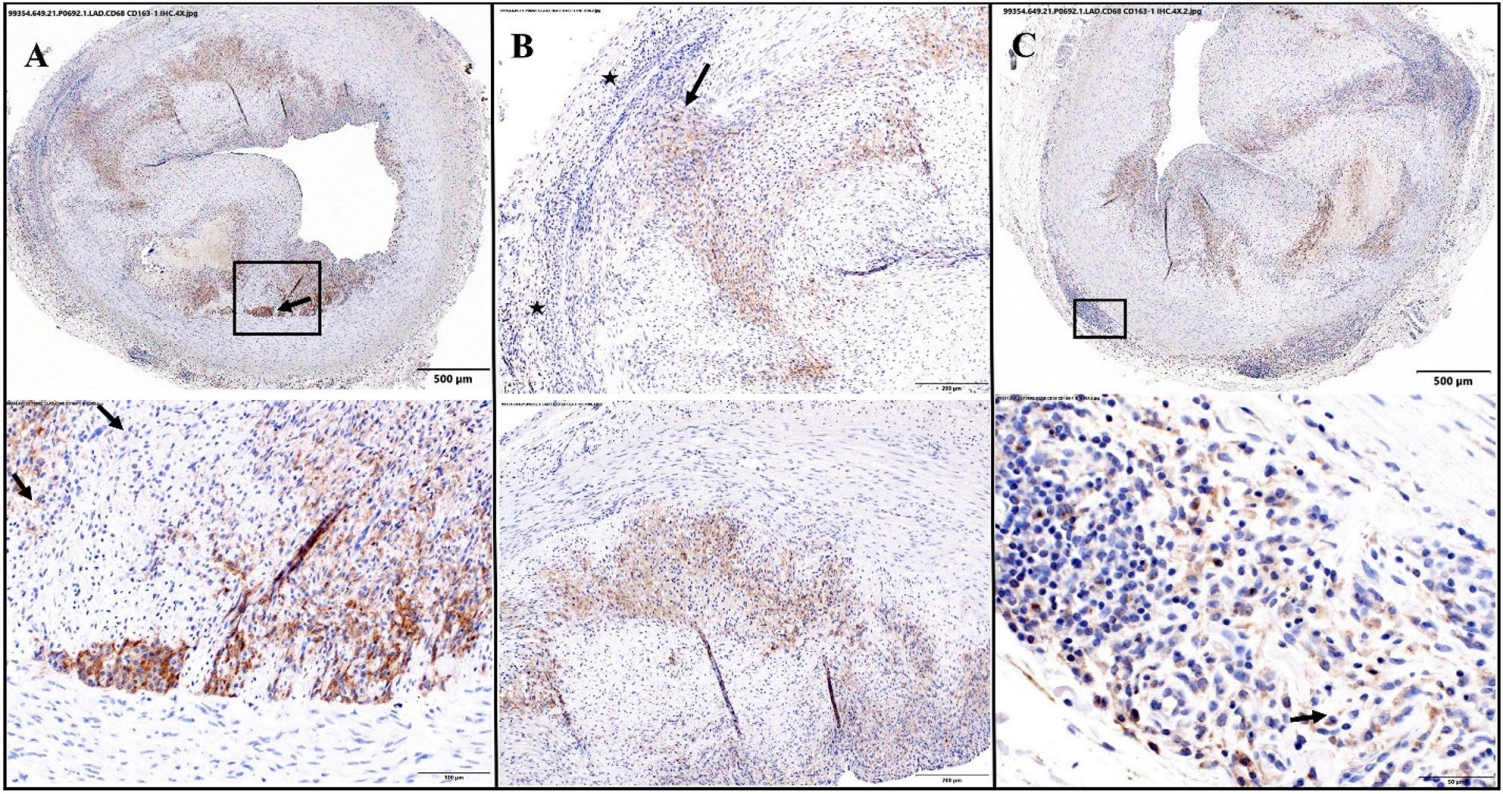
Representative lesion LAD in the FH-WMS: CD68 and CD163-1 IHC, DAB and hematoxylin counterstain. (**A**) The box in the top micrograph denotes the location of the bottom micrograph and is centered on the interface of the intima and tunica media. There is marked intimal hyperplasia. Most cells staining positively for CD68/CD163-1 are within the hyperplastic intima, but there are focal areas in which positively staining dells are within the tunica media (arrow); (**B**) The micrographs show two segmental areas of the arterial wall in which there is marked intimal hyperplasia. The brown staining cells are positive for CD68/CD163-1, markers for macrophages. Most positively staining cells are within the hyperplastic intima. There is a focal area of positive staining in the tunica media (top arrow), and there also is positive staining with hypercellular areas of the adventitia (top stars); and (**C**) The box in the top micrograph denotes the location of the bottom micrograph and is centered on a focal area of hypercellularity of the tunica adventitia. Within the hypercellular area there are cell staining positively for CD68/CD163-1, markers for macrophages. Positive staining cells are also easily discernible in the hyperplastic intima (top).

The coronary segments exhibiting atherosclerotic changes had easily discernible intimal hyperplasia. CD61/CD163-1 positive staining cells were common within these regions and the distribution was not uniform as the density of positive cells varied throughout the intimal hyperplasia around the circumference of the affected artery. The CD61/CD163-1 positive staining cells appeared to be at least slightly more prominent near the IEL as compared to regions near the luminal surface. Commonly, the CD61/CD163-1 positive staining extended into the tunica media in focal areas contiguous with regions of positive staining in the intimal hyperplasia. In some of the atherosclerotic segments, segmental areas of minimal to mild hypercellularity of the adventitia contained CD61/CD163-1 positive cells, which were in a higher density than the native, positive staining cells observed in regions of normal adventitia (Fig. 4).

### Differentially expressed miRNAs

**Figure 5:**
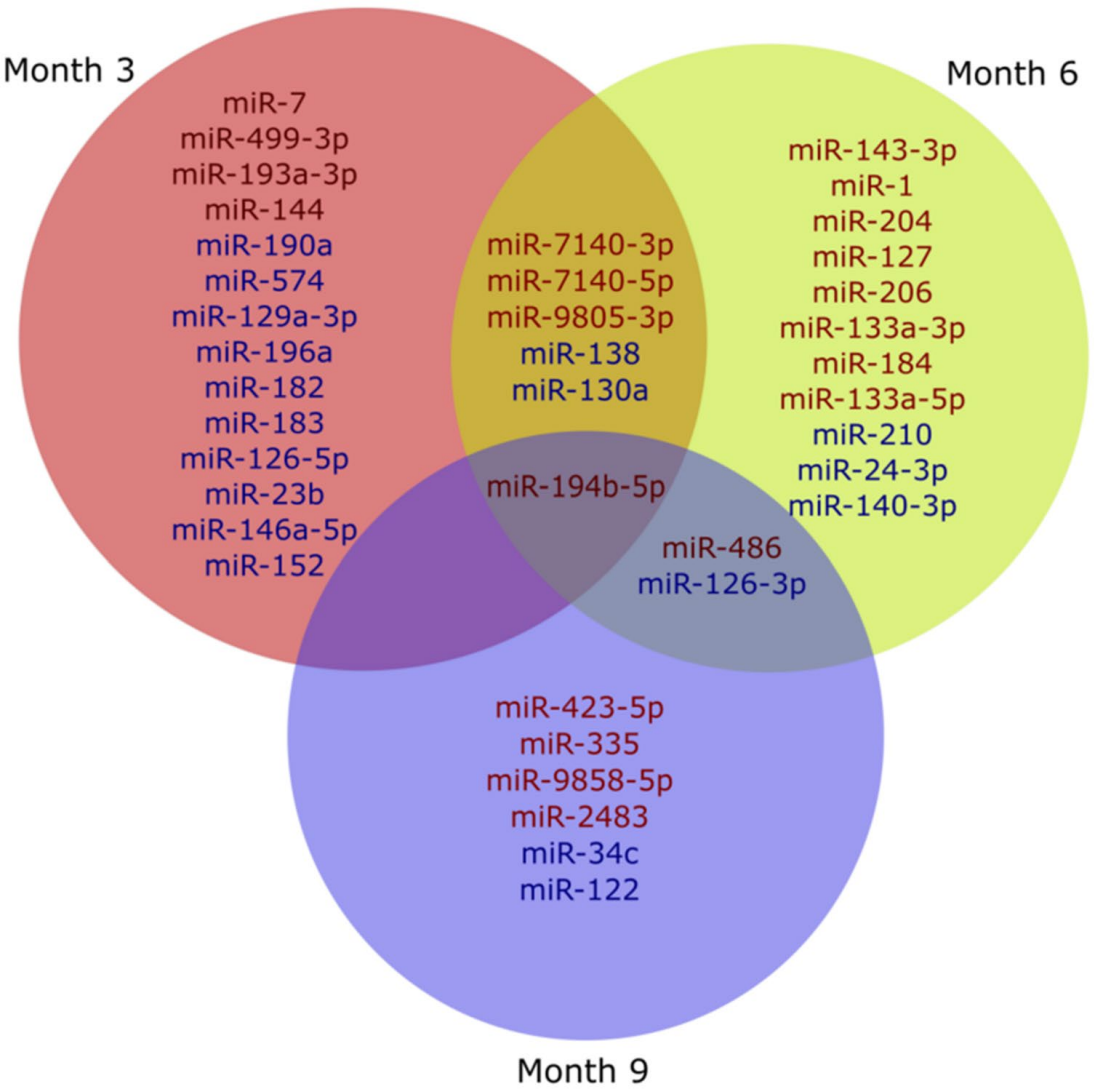
Venn diagram of differentially expressed miRNAs between WMS-FH and WMS-N, at months 3, 6, and 9. Red miRNAs are upregulated in WMS-FH. Blue miRNAs are downregulated in WMS-FH.

We assessed the differential expression of plasma miRNAs between WMS-FH and WMS-N at months 3, 6, and 9. Supplemental Table 3 summarizes the significant DE miRNAs with *p* < 0.1. A total of 20, 19, and 9 DE miRNAs were identified for months 3, 6, and 9, respectively. In addition, some DE miRNAs were present in at least two-time points (i.e., 3 and 6 months, 6 and 9 months, 3 and 9 months, Fig. 5). miR-194b-5p was differentially expressed between WMS-FH and WMS-N at all three-time points (months 3, 6, and 9). In contrast, miR-7140-5p, miR-7140-3p, miR-9805-3p, miR-138, and miR-130a were only captured at months 3 and 6. Similarly, miRNAs miR-486 and miR-126-3p were differentially expressed at two-time points (months 6 and 9) only.

### miRNA targets and gene ontology

miRNAs are known regulators of mRNA expression^16^. We identified validated mRNA targets for the DE miRNAs that share > 90% sequence similarity with human miRNAs (Supplemental Table 4). Several of the obtained mRNAs are targeted by multiple DE miRNAs. Among the targets, we identified chemokines (e.g., CXCL12, CXCL8, CXCR4), cytokines (e.g., CSF1), adhesion molecules (e.g., ICAM1, and VCAM1), growth factors (e.g., VEGFA), integrins (e.g., ITGB1 and 4, ITGA5 and 6), matrix metalloproteinases (MMP13, MMP14, MMP2, MMP7, MMP9), transcription factors (e.g., PPARG, SOX4, SOX9, SOX2, SP1, and YY1), calcium binding proteins (S100A1, S100A8, and S100A9), toll-like receptors (e.g., TLR2, TLR4), and epigenome modifiers (e.g., DNMT1, DNMT3A, SIRT, EP300, MECP2 and SIRT1). The full list of validated targets can be found in Supplemental Table 5. The biological relevance of DE miRNAs was assessed via gene ontology search. Several relevant GO terms to hypercholesterolemia and atherosclerosis were enriched in our results such as negative regulation of interleukin-10 production (miR-194b-5p), negative regulation of amyloid beta clearance (miR-7), positive regulation of cell molecule adhesion (miR-144), positive regulation of high-density lipoprotein particle clearance (miR-144), negative regulation of cholesterol storage and IL-6 and IL-8 production (miR-146a-5p), positive regulation of phagocytosis, cholesterol and fatty acid biosynthesis processes (miR-183-5p), and regulation of vascular associated smooth muscle cell proliferation and differentiation (miR-182). The full data for GO terms per DE miRNA is summarized in Supplemental Table 6. Some of the GO terms were represented by more than one miRNA (Supplemental Table 7).

### RT-qPCR validation of DE miRNAs in the second cohort of animals

We validated a subset of DE miRNAs in a new cohort of animals. Among the tested miRNAs, miR-194b-5p was differentially expressed between WMS-FH and WMS-N at 3, 6, and 9 months of age; however, the RT-qPCR showed a significantly higher expression in WMS-FH at months 6 and 9 but not at month 3. Also, miR-194b-5p expression increased with age (month 6: FC = 1.579, *p* = 0.007; month 9: FC = 3.313, *p* = 0.042). Moreover, DE miR-194b-5p showed expression differences between females and males (*p* = 0.036) at month 9, where females exhibited higher expression for this miRNA. Similar to miRNA sequencing results, upregulated miR-206, downregulated miR-138, and miR-126-3p were also validated at month 6. No statistically significant differences between WMS-FH and WMS-N were found for miR-138 at month 3. Supplemental Table 8 summarizes gene expression of tested miRNAs in the second cohort of animals.

### Diagnostic power of DE miRNAs

**Figure 6:**
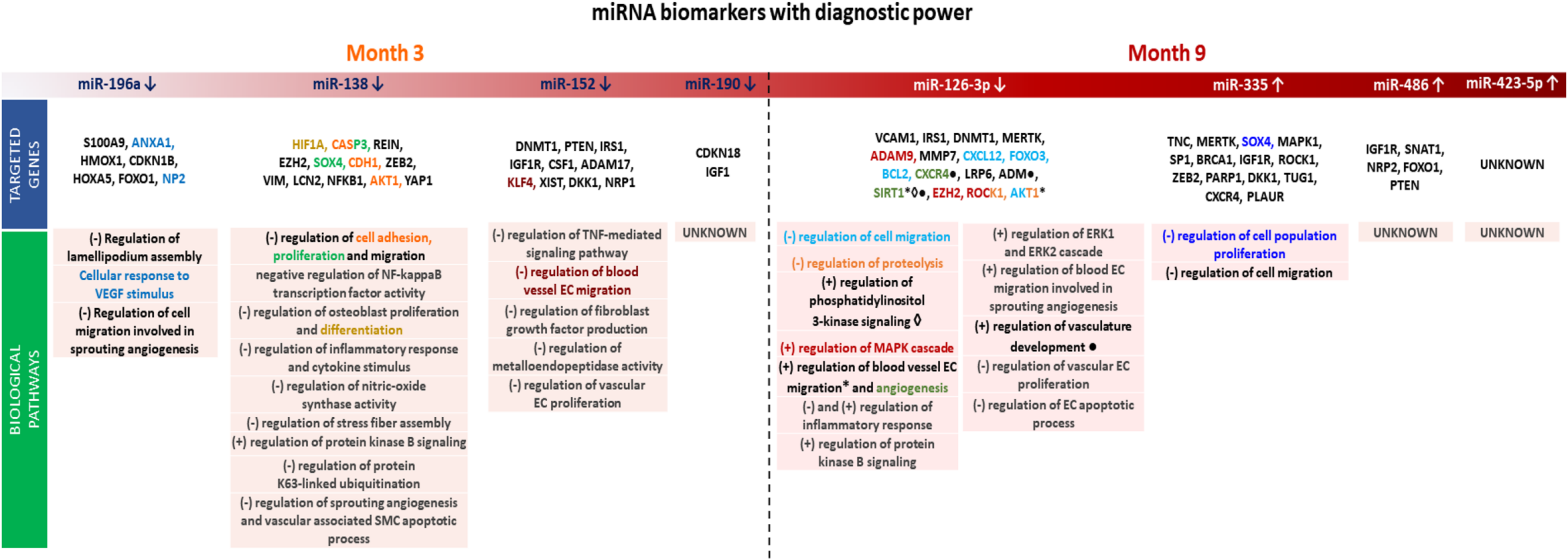
Biological processes associated with those differentially expressed miRNAs with diagnostic power and target genes. The indicated genes in the figure are known to be involved/associated with atherosclerosis development in humans (data obtained from NCBI). Genes participating in a biological process are highlighted in the same color/sign.

The discriminatory power between healthy animals and those with atherosclerotic disease was assessed using the ROC method. Only miRNAs with an AUC threshold that conferred statistical power ≥ 0.8 and *p* < 0.05 were considered powerful enough to distinguish between groups. The estimated AUC threshold was 89.63%. miRNAs that can discriminate between animals with the disease and healthy ones were found at months 3 and 9 (Table 3 and Supplemental Fig. 1), whereas miRNAs at month 6 showed weak discriminatory power. Figure 6 summarizes the biological processes and mRNA targets for those miRNAs with good diagnostic power.

**Table 3:** miRNAs with discriminatory power to differentiate case (disease) from control (healthy). P-value < 0.05.

Age	miRNA	AUC (%)	CI 95%
	Power = 0.8; AUC threshold = 89.63%		
Month3	miR138	90.48	70.68–100
	miR190a	90.48	70.90–100
	miR152	90.48	70.90–100
	miR196a	97.62	91.02–100
Month9	miR423-5p	92.86	77.52–100
	miR486	90.48	70.90–100
	miR335	90.48	73.65–100
	miR126-3p	95.24	84.19–100
	Power = 0.95; AUC threshold = 93.91%		
Month3	miR196a	97.62	91.02–100
Month9	miR126-3p	95.24	84.19–100

## Discussion

Early atherosclerosis detection is a critical step towards the prevention of disease progression and acute plaque destabilization. Several studies have proposed the use of circulating miRNAs as biomarkers to diagnose the presence of atherosclerosis in humans^5,9,10,17^. miRNAs regulate gene expression and play a role in cell-to-cell communication^16^ and are found circulating in the blood bound to lipoproteins^18^ or encapsulated in exosomes or microvesicles^8^. In the present study, we used a well-characterized swine model of atherosclerosis (WMS-FH) to study the DE of circulating miRNAs over a period of 9 months and correlated these changes with the histological presence of atherosclerosis. The study findings show that (a) atherosclerosis was diagnosed by in vivo multimodality imaging and confirmed by histopathology in 7/9 (78%) of the WMS-FH and in none of the animals in the control group, (b) several DE miRNAs were identified at multiple time points and few remained DE overtime, (c) some of these DE miRNAs showed decent discriminatory power for the diagnosis of coronary artery atherosclerosis and (d) the gene targets and biological processes influenced by these miRNAs provided insight into their possible biological relevance in the context of atherosclerosis development.

Our study demonstrated that circulating miRNA profiles start to differ between WMS-FH and WMS-N at very early stages of atherosclerosis development and continue to change over time. Only a handful of miRNAs remained DE over time, demonstrating the complexity and variability of the biological processes involved in atherosclerotic lesions. Our results indicate that DE miRNAs undergo dynamic changes in the early stages of atherosclerosis and continue to be dysregulated in the advanced stages of disease development. These miRNAs appear and reappear over time depending on the biological state of the disease and the involved mechanisms. This is supported by the detection of DE miR-194b-5p at months 3, 6, and 9 and other miRNAs that were captured only at one time point in the present work.

The nature of the DE miRNAs changes seen over time supports some of the well-described biological mechanisms involved in atherosclerosis development. At 3 months, many of the DE miRNAs regulate biological processes involving cell adhesion, migration, and proliferation. Regulation of cellular cholesterol clearance and foam cell formation were predominant at this timeframe. Among the DE miRNAs found in our study, miR-7, miR-144, miR-146a-5p, and miR-24-3p regulate foam cell formation. miR-7 and miR-144 were upregulated and promote foam cell formation by directly downregulating genes involved in cholesterol efflux (STK11 and TR4)^19,20^. In contrast, miR-146a and miR-146-5p were downregulated in the WMS-FH at 3 months. Specifically, miR-146-5p appears to have an atheroprotective effect by preventing foam cell formation through the downregulation of NF-κB and TRAF6 expression^21,22^.

DE miRNAs involved in angiogenesis and smooth muscle cell proliferation were predominant at 6 months. miRNAs involved in the regulation of smooth muscle cell dynamics, including regulation of phenotypic switching, actin cytoskeleton organization and activation of protein kinase B activity were particularly prominent. In our study, miR-23b, miR-206, miR-204, miR-1, and miR-34c, known to regulate smooth muscle cell proliferation, were differentially expressed. miR-23b and miR-34c were downregulated in WMS-FH and have been shown to tightly regulate SMC proliferation and prevent neointima formation by targeting LRP6^29^ and SCF^23^. Another downregulated miRNA in WMS-FH is miR-182 which was shown recently to inhibit vascular smooth muscle cell proliferation^24^. Similarly, miR-24 was shown to inhibit endothelial cell proliferation^25^. Moreover, reduced expression of miR-24 resulted in increased MMP14 expression and lesion size in mice^26^.

The only miRNA upregulated across the three time points in the WMS-FH was miR-194b-5p. This miRNA targets IL-10 and is involved in its negative regulation (Supplemental Table 5). IL-10 has been shown to confer protection against atherosclerosis^27,28^. Therefore, the consistent upregulation of this miRNA indicates another lacking mechanism of atheroprotection in WMS-FH that is persistent and spans a long period. However, the proatherogenic role of miR-194b-5p remains to be investigated.

Several DE miRNAs found in our study have been reported in human studies of atherosclerosis. For example, the downregulation of miR-574 in WMS-FH was negatively associated with the presence of atherosclerosis. In the Framingham Heart Study, miR-574-3p correlated negatively with stroke^9^. Similarly, miR-182 and miR-152 expression levels were lower in patients with coronary artery atherosclerosis^24,29^. Additionally, downregulated miR-130a in WMS-FH correlated negatively with the presence of atherosclerosis^30^. miR-130a and miR-210 were found to be downregulated in the WMS-FH, these miRNA levels have been found elevated in the serum of patients with peripheral artery atherosclerosis^31^. Due to the early stage of disease development, our model displayed lesions in the coronary arteries only; hence, some of these differences may be related to the stage of disease development. Several studies have shown that circulating miRNA signatures differ based on atherosclerosis location^32,33^. Then, it is also possible that differences in miRNA expression may be related to differences in developmental stage and disease location.

At 3 months, miRNA-138, miR-152, miR-196a, and miR-190a were downregulated and had the best diagnostic discriminatory power to detect coronary atherosclerosis. miR-138 has been described to be atheroprotective by reducing coronary endothelial cell injury^34^ and regulating endothelial dysfunction^35^. Similarly, miR-152 exhibited atheroprotection by targeting KLF5 and reducing inflammation in mice^36^ and its expression levels were lower in patients with coronary artery atherosclerosis^24,29^. miR-196a showed the strongest diagnostic power at 3 months, and it has been reported to have low expression in patients with myocardial infarction^37^. Downregulation of miR-196a in the plasma of patients with familial hypercholesterolemia has been linked to its strong diagnostic power in early atherosclerosis detection^38^. miR-190a has not well described biological function or role in atherosclerosis.

There were no DE miRNAs with discriminatory power at 6 months. At 9 months miR-486-5p, miR-335-5p, miR-126-3p, and miR-423-5p had the highest diagnostic discriminatory power to detect coronary atherosclerosis. miR-486-5p was upregulated and reported to target HAT1, which then downregulates ABCA1, hence, affecting cholesterol efflux^47^; thus, it promotes foam cell formation. miR-486 has been also shown to regulate endothelial cell function and inflammatory processes associated with atherosclerosis^39^. Likewise, miR-335-5p was upregulated and inhibits macrophage immune responses by targeting Notch signaling resulting in reduced atherosclerotic vulnerable plaque formation^40^. miR-126-3p was significantly downregulated in WMS-FH compared to WMS-N controls and it carried a strong diagnostic power for the presence of coronary artery atherosclerosis at 9 months. Experimental studies suggest that miR-126-3p plays a significant role in endothelial function, inflammation, and signaling pathways relevant to atherosclerosis by modulating mechanisms such as the mitogen-activated protein kinase (MAPK) cascade, and the phosphatidylinositol-3-kinase (PI3K) signaling pathway^41^ and targeting key mediators such as VCAM-1, IL-6, and TNF-α^42^. In humans, miR-126-3p expression levels were shown to distinguish between individuals with and without coronary atherosclerosis^43^. Moreover, this miRNA was significantly lower in patients with coronary artery atherosclerosis compared to healthy patients^44^. miR-423-5p does not have a well-described biological function or role in atherosclerosis.

miR-196a is the miRNA with the highest discriminatory power and earliest detection (month 3) in the present study. Few studies have explored its role in atherosclerosis development^45–47^. An unexplored mechanism captured in our data is the regulation of lamellipodium assembly that appears to be targeted by miR-196a. Lamellipodium assembly plays a crucial role in cell migration^48^, a process that is heavily involved in atherosclerosis. Also, our findings suggest an association between miR-138 and K63-linked ubiquitination, a regulatory mechanism associated with inflammation, cell survival, and oxidative stress^49^*.* In addition, miR-335 was shown to regulate the proliferation and smooth muscle cells phenotypic switching through direct modulation of SP1^50^. The relevance of this mechanism in atherosclerosis is yet to be established and warrants further investigation. A remarkably interesting mechanism is the negative regulation of amyloid beta clearance that appears to be associated with DE miR-7. Aberrant deposition and clearance of amyloid from the subintima have been linked to inflammation and the promotion of atherosclerosis^51^. Also, miR-138 plays a role in the regulation of osteoblast differentiation^52^, suggesting that vascular calcification starts early in the process of atherosclerosis development.

Finally, several DE miRNAs were found to target epigenetic modifiers such as DNMT1, DNMT3A, and EP300, suggesting the presence of alterations in epigenetics mechanisms between WMS-FH and WMS-N. Alterations in epigenetic marks result in aberrant gene expression and phenotypes^53^. Furthermore, aberrations in epigenetic marks have been linked to atherogenesis^53–56^. Some of the miRNAs found in this study (miR-7140-3p, miR-7140-5p, miR-9805-3p, miR-9858-5p, and miR-2483) did not share similarities with any known miRNA human, mice, rat, rabbits, or non-human primate sequences currently available in miRBase. The uniqueness of these miRNAs suggests that they may be swine-specific. However, these miRNAs may also be novel ones that are yet to be identified. Nonetheless, these miRNAs deserve further investigations to assess their role in atherosclerosis.

One limitation of the present work is that GO search was carried out for those miRNAs similar in sequence to human miRNAs. Another limitation was the absence of live imaging in animals at each studied time point to confirm the absence of the disease in early life. However, WMS-FH swine develop lesions in the arteries around 1-year-old when the animals are fed the normal diet used in this work. Also, the work focused on early atherosclerosis, which is confirmed with OCT imaging and histopathology that indicates the absence of advanced stages of the disease in FH animals. Also, our study is limited to miRNA profiles at months 3, 6 and 9 of age. Profiling earlier time points than month 3, could potentially provide earlier biomarkers for atherosclerosis detection. However, we have opted for the time points for the following reasons. (1) WMSH FH show a peak in cholesterol levels around month 6 of age. (2) The total cholesterol levels stabilize around month 9. (3) Month 3 was selected a time point that allows for similar intervals of time for profiling. Additionally, we observe lesions in this model typically around the age of 12 months with no lesions observed at month 9, 6 or 3.

## Conclusion

The findings above demonstrate that circulating miRNAs expression differs between WMS-FH and WMS-N, and these differences can be identified in animals’ early life before the manifestation of atherosclerosis. Additionally, these miRNAs are involved in mechanisms governing the pathophysiology of the disease, further supporting the evidence of early onset before disease manifestation; hence, these miRNAs are promising biomarkers for early detection. Specifically, in WMS-FH, atherosclerosis can be detected at 3 months of age using miR-138, miR-152, miR-190a, and miR-196a. While miRNAs miR486, miR-126-3p, miR-335, and miR-423-5p can detect the disease at month 9 of age.

## Methods

The study was approved by the Institutional Animal Care and Use Committees of the the University of Wisconsin-Madison (Madison, WI) and CRF Skirball Center of Innovation (Orangeburg, NY). All animals received standard care in compliance with the *Guide for the Care and Use for Laboratory Animals*^57^ and the Animal Welfare Act^58^*.* All methods herein are reported in accordance with ARRIVE guidelines (https://arriveguidelines.org). The methodology followed in the present study is summarized in Fig. 1.

### Experimental model

Two cohorts of the WMS-FH and WMS normal (WMS-N) were selected for this study. WMS-N pigs have the same genetic background as WMS-FH; however, these swine are normocholesterolemic with a completely functional LDLR. Cohort 1 had nine WMS-FH (females) and nine WMS-N (females). In the second cohort, 15 WMS-FH (females n = 8, males n = 7) and 15 WMS-N (females n = 8, males n = 7) were included. Animals were randomly selected and housed with the main herd at the Swine Research and Teaching Center (SRTC) at the University of Wisconsin-Madison throughout the duration of the study. All animals were fed the same diet formulated to meet the nutritional requirements of swine (Supplemental Table 1). Cohort 1 animals were used to profile plasma cell-free RNAs using small-RNA sequencing and identify DE miRNAs, whereas cohort 2 animals were used for biological validation of RNA-Sequencing results using RT-qPCR.

### Multi-modality imaging analysis

#### Quantitative coronary angiography analysis

At 12 months, Quantitative Coronary Angiography analysis (QCA) was performed using QAngio XA SoftwareTM 7.1.14.0 (Medis Medical Imaging System, Leiden, The Netherlands). A contrast-filled catheter was used for calibration. The minimum lumen diameter (MLD) was obtained from a single view with the lowest measurement, while the reference vessel diameter (RVD) was automatically calculated. Percent diameter stenosis (%DS) was calculated from the MLD and the RVD.

#### Intravascular imaging analysis

Intravascular Ultrasound (IVUS) pullback images were generated (Atlantis® SR Pro 40 MHz catheters and iLab system, Boston Scientific, Natick, MA) and analyzed with a commercially available software (echoPlaque, Indec Systems Inc., Santa Clara, CA). Luminal- and vessel areas were measured, and the plaque burden and plaque volume were calculated. Optical Coherence Tomography (OCT) images were obtained using the ILUMIEN OPTIS imaging system (St. Jude Medical, St. Paul, MN). Qualitative analyses were performed at 1-mm intervals with the commercial software (ILUMIEN OPTIS, Version E.4). Cross-section lumen, vessel areas and intimal thickness were measured, and percent area stenosis was calculated as (vessel area-lumen area)/vessel area * 100%.

### Blood collection and plasma processing

Whole blood was collected longitudinally at 3, 6, and 9 months from each animal in 8 mL K3 EDTA tubes (Covidien, MA, USA). Blood tubes were centrifuged at 4 °C for 10 min to separate plasma. Next, collected plasma was centrifuged using the same settings to remove any cellular debris. Samples were then preserved in 1.5 mL tubes at − 80 °C until needed. The three-time points were selected based on the cholesterol profiles of WMS-FH pigs. Month 3 was selected as an early time point before serum cholesterol levels peak and stabilize. Total cholesterol then peaks around month 6 and stabilizes around month 9.

### Hemolysis and total cholesterol measurements

Studies have shown that hemolysis considerably affects the levels of cell-free miRNAs^59^. To correct for this effect, we measured hemolysis in plasma samples by assessing absorbance at A414 using NanodropONE spectrophotometry (Thermofisher Scientific, DE, USA). For each sample, the average of at least three measurements was calculated. The A414 absorbance values were used as a covariate in statistical analysis to assess the differential expression of miRNAs. Total cholesterol levels were measured in plasma samples using ELISA Cholesterol Assay Kit—HDL and LDL/VLDL (Abcam, CA, USA) following the product’s manual. Plasma samples from WMS-FH were diluted (twofold) so that colorimetric readings fell within the range of the cholesterol standard curve.

### miRNA extraction

Cell-free RNA (cf-RNA) was extracted from plasma samples using Quick-cfRNA Serum & Plasma Kit (Zymo Search, CA, USA). Briefly, plasma samples were centrifuged at 12,000 g for 15 min to remove cellular debris, then 400 uL of plasma was digested for 2 h at 37 °C using proteinase K. After digestion, the remaining steps were followed as described in the manufacturer’s manual. To obtain concentrated samples, we eluted the column content in 10-uL of nuclease-free water. For biological validation samples used in RT-qPCR (cohort 2 of animals), cel-miR-39 mimic (QIAGEN, CA, USA) was spiked in each sample after 2 h of digestion to assess extraction efficiency and consistency across samples.

### Small RNA sequencing and miRNA differential expression analysis

Small RNA sequencing was performed by RealSeq-Biosciences (CA, USA). Library preparation was carried out using the RealSeq-Biofluids library prep kit (RealSeq Biosciences). Libraries were then sequenced on a NextSeq 500 v2 High-Output with a read length of 75 bp in one direction (single read). Both raw and trimmed fastq files were obtained from RealSeq-Biosciences. For each file, FastQC^60^ was used to assess sequencing quality. We performed bioinformatics analysis using OASIS for small RNA sequencing pipeline^61^. Briefly, trimmed reads were aligned to the swine miRNA database and reference genome (Sus Scrofa ssc3) using STAR^62^. Next, using DESeq2^63^, the obtained mapped raw reads were normalized, and differential expression was performed following the negative binomial generalized linear regression model. The regression model included hemolysis in plasma samples, weight of animals and grouping (WMS-FH vs. WMS-N) for each time point (M3, M6, or M9). Time course analysis to detect changes in miRNA expression within individual animals and between groups included the age of animal as an additional variable (M3, M6, or M9). Only miRNAs with adjusted *p* value < 0.1 were considered significantly differentially expressed between WMS-FH and WMS-N.

### miRNA target prediction and gene ontology

To assess the biological function of the differentially expressed miRNAs, we predicted the miRNA targets and miRNA functions via Gene Ontology (GO). Swine differentially expressed miRNA sequences were blasted against the human miRNAs using the miRBase database^16^ to check for sequence similarity. Human miRNAs that are (> 90%) similar in sequence to the swine miRNAs were used to search for mRNA targets available in the database of validated miRNA-target interactions (miRTarBase)^64^. GO terms for each miRNA were obtained using miRBase database^65^.

### Validation of DE miRNAs via RT-qPCR

We selected a subset of DE miRNAs for validation in a new cohort of animals. Selection criteria were based on expression pattern (up/downregulation), early time point of detection (month 3 or 6), and detection at multiple time points (months 3, 6, and 9). A total of 30 samples (cohort 2) were used to validate DE miRNAs. For each plasma sample, 2-uL of extracted cf-RNAs were used in cDNA synthesis with MIRCURY LNA RT kit (QIAGEN). Additionally, each cDNA reaction was spiked in with UniSp6 to assess a consistent cDNA synthesis across all samples. MIRCURY LNA assay primers were obtained from QIAGEN. MIRCURY LNA SYBR Green assay kit (QIAGEN) was used to assess the expression of miRNAs. Also, two miRNA endogenous controls (bta-miR-93 and miR-17-3p) were used to normalize expression results. The endogenous controls were selected based on the stability of multiple miRNAs using Normfinder^66^. miR-17-3p showed high stability in all sequenced samples. cf-RNA extraction was repeated for samples showing variability of cel-miR-39 and UniSp6 expression. The common base method^67^ was used to assess differential expression, where each well of the RT-qPCR assay efficiency was incorporated in the final calculation of the ΔΔCT method^68^.

### Histopathology evaluation

Representative samples of the proximal to mid regions of the right coronary artery (RCA) and/or the left anterior descending coronary artery (LAD) were taken for histology following fixation consisting of perfusion of the coronary vasculature with 1 L of 10% neutral buffered formalin after initial clearance of blood with 1 L of normal saline and then additional immersion fixation in 10% NBF for a minimum of 24 h. The arterial segments were processed by routine methods and embedded in paraffin. The resulting paraffin blocks were cut at 5-μm thickness and immunohistochemically stained for CD68 and CD163-1 and examined for the presence, relative amount, and distribution of positive staining. Monoclonal antibodies for CD68 (M1-type macrophage marker) and CD163-1 (M2-type macrophage markers) were used simultaneously with the secondary antibody (mouse-on-FARMA-HRP (horseradish peroxidase) and diaminobenzamide (DAB) was used as a chromogen) detecting both primary antibodies with no differentiation between the two; therefore, the immunohistochemical assay was designed to detect all macrophages within the examined tissue without differentiating between the 2 subtypes (panmacrophage IHC). Lymph nodes from swine served as a positive control. Sections of the same lymph nodes were stained with the same procedure using normal mouse IgG as the primary antibody to serve as a control for the assessment of nonspecific staining. The IHC-stained sections were evaluated for the presence of the DAB chromogen. The amount of positive staining was semiquantitatively scored using standard methods. The control section from the full staining protocol showed intensely positive staining cells in regions of the lymph nodes consistent with macrophage populations; therefore, the IHC assay performed well in detecting tissue macrophages. The sections were counterstained with H&E. The primary control section showed no discernible staining; therefore, there was no discernible nonspecific binding by the secondary antibody. The IHC stain employed in this investigation was not designed to allow distinction of the details of tissue architecture, as the tissue stains were only the DAB chromogen of the CD61/CD163-1-positive cells and a hematoxylin counterstain. However, the presence of certain specific features including intimal thickening/hyperplasia, cellular density, and characteristics consistent with atherosclerosis could be distinguished, and their association with the CD61/CD163-1 positive staining was noted when present.

### Discriminatory power analysis

To determine the diagnostic power of miRNAs, we performed a discriminatory power analysis using the ROC curves of the pROC R package^69^. First, animals were categorized into “healthy” and “with disease” based on imaging and histo-pathology results. Only significantly DE miRNAs obtained with DESeq2 from each time point were used in this step. Next, we used normalized sequencing reads for each miRNA to measure the area under the curve (AUC), the confidence interval, and significance level. Due to the small sample size, we determined those miRNAs with AUCs that show a statistical power of 0.95 and 0.8 with α = 0.05.

### Statistical analysis

A two tailed t test was used to assess significant differences in animal characteristic between groups. For miRNA sequencing data, DE miRNAs and statistical significance were determined using a negative binomial regression model that included the following covariables: hemolysis, weight, and group (WMS-FH vs. WMS-N). For RT-qPCR gene expression, a general linear regression model was used to determine the statistical significance in miRNA differences between WMS-FH and WMS-N with animal weight, plasma hemolysis, and sex of animals as variables using the R programming language^70^. Finally, the statistical significance of the AUC was estimated using *power.roc.test* function in pROC package^69^.

### Supplementary Information

Supplementary Information.

## Data Availability

Raw sequencing data generated in this study can be found on NCBI GEO using ID: GSE242074 and BioProject PRJNA1011297. Alternatively, the data can be accessed using this link (https://www.ncbi.nlm.nih.gov/geo/query/acc.cgi?acc=GSE242074).
