# Supplementary material for: Longitudinal dynamics of circulating miRNAs in a swine model of familial hypercholesterolemia during early atherosclerosis
Source: Sci Rep. 2023 Nov 7;13:19355. doi: 10.1038/s41598-023-46762-0 (PMC10630391; doi:10.1038/s41598-023-46762-0)
Supplement: Supplementary file 1 — Supplementary Information. [file 41598_2023_46762_MOESM1_ESM.docx]

**Table 1.** Composition of the pellet diet fed to WMS FH and WMS normal pigs used in the study.

| **Ingredients** | **Amount (%)** |
| --- | --- |
| Corn grain | 75.51 |
| Soybean meal, (46%) | 21.25 |
| Monocalcium phosphate (21%) | 0.67 |
| Calcium Carbonate (limestone) | 1.09 |
| I-lysine mHCL | 0.12 |
| Sodium chloride (Iodized) | 0.35 |
| UW VTMM04 G | 1.00 |
| **Total** | **100** |

**Table 2 .** Characteristics of the animal cohort used for miRNA sequencing.

| **Variables** | **WMS-FH** | | | **WMS-N** | | |
| --- | --- | --- | --- | --- | --- | --- |
| **Age** ^ns^  (Months, mean ± SD) | Month3 | Month6 | Month9 | Month3 | Month6 | Month9 |
|  | 03.03± 0.08 | 6.06± 0.06 | 9.16± 0.02 | 2.98± 0.08 | 5.97± 0.10 | 9.10± 0.10 |
| **Weight**^ns^  (kg, mean ± SD) | 15.16± 2.32 | 26.73± 5.46 | 38.67± 9.66 | 14.48± 3.21 | 25.19± 4.20 | 42.27± 8.11 |
| **Total cholesterol*****  (mg/dL, mean ± SD) | 281.75± 89.86 | 436.9± 142.95 | 436.77± 238.99 | 81.52±20.44 | 96.78±35.25 | 61.73± 12.44 |

^FH: familial hypercholesterolemia; SD: standard deviation. ***: statistically signficant difference between groups (p<0001) at three time points (months 3, 6, and 9). ns: no statistical significance between groups (p > 0.1).^

**Table 3.** Significantly differentially expressed miRNAs between WMS-FH and WMS-N at months 3, 6, and 9 (p < 0.1).

| **Age** | **miRNA** | **Fold change** | **Adjusted p-value** | **Expression** |
| --- | --- | --- | --- | --- |
| **Month 3** | miR-7140-3p | 3.74 | 0.048 | Upregulated |
|  | miR-7140-5p | 6.28 | 0.048 | Upregulated |
|  | miR-194b-5p | 3.16 | 0.085 | Upregulated |
|  | miR-7 | 1.47 | 0.086 | Upregulated |
|  | miR-499-3p | 1.88 | 0.086 | Upregulated |
|  | miR-193a-3p | 1.34 | 0.086 | Upregulated |
|  | miR-144 | 1.45 | 0.090 | Upregulated |
|  | miR-9805-3p | 2.18 | 0.090 | Upregulated |
|  | miR-183 | 0.64 | 0.048 | Downregulated |
|  | miR-190a | 0.53 | 0.048 | Downregulated |
|  | miR-574 | 0.58 | 0.048 | Downregulated |
|  | miR-129a-3p | 0.66 | 0.053 | Downregulated |
|  | miR-196a | 0.55 | 0.069 | Downregulated |
|  | miR-182 | 0.51 | 0.070 | Downregulated |
|  | miR-138 | 0.61 | 0.070 | Downregulated |
|  | miR-126-5p | 0.69 | 0.086 | Downregulated |
|  | miR-130a | 0.78 | 0.086 | Downregulated |
|  | miR-23b | 0.70 | 0.086 | Downregulated |
|  | miR-146a-5p | 0.74 | 0.086 | Downregulated |
|  | miR-152 | 0.72 | 0.090 | Downregulated |
| **Month 6** | miR-7140-5p | 6.27 | 0.000 | Upregulated |
|  | miR-7140-3p | 5.13 | 0.000 | Upregulated |
|  | miR-143-3p | 1.91 | 0.037 | Upregulated |
|  | miR-9805-3p | 3.74 | 0.041 | Upregulated |
|  | miR-194b-5p | 5.67 | 0.041 | Upregulated |
|  | miR-1 | 10.35 | 0.041 | Upregulated |
|  | miR-204 | 6.20 | 0.041 | Upregulated |
|  | miR-127 | 2.46 | 0.050 | Upregulated |
|  | miR-486 | 1.98 | 0.071 | Upregulated |
|  | miR-206 | 10.72 | 0.084 | Upregulated |
|  | miR-133a-3p | 7.52 | 0.096 | Upregulated |
|  | miR-184 | 3.41 | 0.098 | Upregulated |
|  | miR-133a-5p | 5.63 | 0.098 | Upregulated |
|  | miR-138 | 0.44 | 0.037 | Downregulated |
|  | miR-126-3p | 0.61 | 0.037 | Downregulated |
|  | miR-130a | 0.68 | 0.050 | Downregulated |
|  | miR-210 | 0.55 | 0.054 | Downregulated |
|  | miR-24-3p | 0.71 | 0.098 | Downregulated |
|  | miR-140-3p | 0.57 | 0.098 | Downregulated |
| **Month 9** | miR-486 | 1.81 | 0.015 | Upregulated |
|  | miR-423-5p | 1.30 | 0.015 | Upregulated |
|  | miR-335 | 1.56 | 0.038 | Upregulated |
|  | miR-9858-5p | 1.72 | 0.037 | Upregulated |
|  | miR-194b-5p | 3.42 | 0.071 | Upregulated |
|  | miR-2483 | 1.45 | 0.083 | Upregulated |
|  | miR-34c | 0.46 | 0.037 | Downregulated |
|  | miR-122 | 0.43 | 0.071 | Downregulated |
|  | miR-126-3p | 0.71 | 0.083 | Downregulated |

**Table 4.** DE swine miRNA sequences and the corresponding human miRNA ID and sequence.

| **Swine miRNA ID** | **Swine seq** | **Human miRNA ID** | | **Human Seq** | |
| --- | --- | --- | --- | --- | --- |
| ssc-miR-7140-3p | augaugccccuuagaguugagc | NA | na | |  |
| ssc-miR-7140-5p | caacucaagggggcaucauuca | NA | na | |  |
| ssc-miR-194b-5p | uguaacagcgacuccaugugga | hsa-miR-194-5p | uguaacagcaacuccaugugga | |  |
| ssc-miR-7 | uggaagacuagugauuuuguuguu | hsa-miR-7-5p | uggaagacuagugauuuuguuguu | |  |
| ssc-miR-499-3p | aacaucacagcaagucugugcu | hsa-miR-499a-3p | aacaucacagcaagucugugcu | |  |
| ssc-miR-193a-3p | aacuggccuacaaagucccagu | hsa-miR-193a-3p | aacuggccuacaaagucccagu | |  |
| ssc-miR-144 | uacaguauagaugauguac | hsa-miR-144-3p | uacaguauagaugauguacu | |  |
| ssc-miR-9805-3p | cccagggucugucggaugucu | NA | Na | |  |
| ssc-miR-183 | uauggcacugguagaauucacug | hsa-miR-183-5p | uauggcacugguagaauucacu | |  |
| ssc-miR-190a | ugauauguuugauauauuagg | hsa-miR-190a-5p | ugauauguuugauauauuagg | |  |
| ssc-miR-574 | cacgcucaugcacacacccaca | hsa-miR-574-3p | cacgcucaugcacacacccaca | |  |
| ssc-miR-129a-3p | aagcccuuaccccaaaaagcau | hsa-miR-129-2-3p | aagcccuuaccccaaaaagcau | |  |
| ssc-miR-196a | uagguaguuucauguuguuggg | hsa-miR-196a-5p | uagguaguuucauguuguuggg | |  |
| ssc-miR-182 | uuuggcaaugguagaacucacacu | hsa-miR-182-5p | uuuggcaaugguagaacucacacu | |  |
| ssc-miR-138 | agcugguguugugaaucaggc | hsa-miR-138-5p | agcugguguugugaaucaggc cg | |  |
| ssc-miR-126-5p | cauuauuacuuuugguacgcg | hsa-miR-126-5p | cauuauuacuuuugguacgcg | |  |
| ssc-miR-130a | cagugcaauguuaaaagggcau | hsa-miR-130a-3p | cagugcaauguuaaaagggcau | |  |
| ssc-miR-23b | aucacauugccagggauuacca | hsa-miR-23b-3p | aucacauugccagggauuacca c | |  |
| ssc-miR-146a-5p | ugagaacugaauuccauggguu | hsa-miR-146a-5p | ugagaacugaauuccauggguu | |  |
| ssc-miR-152 | ucagugcaugacagaacuugg | hsa-miR-152-3p | ucagugcaugacagaacuugg | |  |
| ssc-miR-7140-5p | augaugccccuuagaguugagc | NA | na | |  |
| ssc-miR-7140-3p | caacucaagggggcaucauuca | NA | na | |  |
| ssc-miR-143-3p | ugagaugaagcacuguagcuc | hsa-miR-143-3p | ugagaugaagcacuguagcuc | |  |
| ssc-miR-9805-3p | cccagggucugucggaugucu | NA | na | |  |
| ssc-miR-194b-5p | uguaacagcgacuccaugugga | hsa-miR-194-5p | uguaacagcaacuccaugugga | |  |
| ssc-miR-1 | uggaauguaaagaaguaugua | hsa-miR-1-3p | uggaauguaaagaaguaugua u | |  |
| ssc-miR-204 | uucccuuugucauccuaugccu | hsa-miR-204-5p | uucccuuugucauccuaugccu | |  |
| ssc-miR-127 | ucggauccgucugagcuuggcu | hsa-miR-127-3p | ucggauccgucugagcuuggcu | |  |
| ssc-miR-486 | uccuguacugagcugccccgag | hsa-miR-486-5p | uccuguacugagcugccccgag | |  |
| ssc-miR-206 | uggaauguaaggaaguguguga | hsa-miR-206 | uggaauguaaggaagugugugg | |  |
| ssc-miR-133a-3p | uugguccccuucaaccagcug | hsa-miR-133a-3p | u uugguccccuucaaccagcug | |  |
| ssc-miR-184 | uggacggagaacugauaagggu | hsa-miR-184 | uggacggagaacugauaagggu | |  |
| ssc-miR-133a-5p | agcugguaaaauggaaccaaau | hsa-miR-133a-5p | agcugguaaaauggaaccaaau | |  |
| ssc-miR-138 | agcugguguugugaaucaggc | hsa-miR-138-5p | agcugguguugugaaucaggc cg | |  |
| ssc-miR-126-3p | ucguaccgugaguaauaaugcg | hsa-miR-126-3p | ucguaccgugaguaauaaugcg | |  |
| ssc-miR-130a | cagugcaauguuaaaagggcau | hsa-miR-130a-3p | cagugcaauguuaaaagggcau | |  |
| ssc-miR-210 | cugugcgugugacagcggcuga | hsa-miR-210-3p | cugugcgugugacagcggcuga | |  |
| ssc-miR-24-3p | uggcucaguucagcaggaacag | hsa-miR-24-3p | uggcucaguucagcaggaacag | |  |
| ssc-miR-140-3p | uaccacaggguagaaccacggac | hsa-miR-140-3p | uaccacaggguagaaccacgg minus ac | |  |
| ssc-miR-486 | uccuguacugagcugccccgag | hsa-miR-486-5p | uccuguacugagcugccccgag | |  |
| ssc-miR-423-5p | ugaggggcagagagcgagacuuu | hsa-miR-423-5p | ugaggggcagagagcgagacuuu | |  |
| ssc-miR-335 | ucaagagcaauaacgaaaaaug | hsa-miR-335-5p | ucaagagcaauaacgaaaaaug u | |  |
| ssc-miR-9858-5p | uuccugagucggacugggcu | NA | na | |  |
| ssc-miR-194b-5p | uguaacagcgacuccaugugga | hsa-miR-194-5p | uguaacagcaacuccaugugga | |  |
| ssc-miR-2483 | aaacaucugguugguugagaga | NA | na | |  |
| ssc-miR-34c | aggcaguguaguuagcugauugc | hsa-miR-34c-5p | aggcaguguaguuagcugauugc | |  |
| ssc-miR-122 | uggagugugacaaugguguuugu | hsa-miR-122-5p | uggagugugacaaugguguuug minus u | |  |
| ssc-miR-126-3p | ucguaccgugaguaauaaugcg | hsa-miR-126-3p | ucguaccgugaguaauaaugcg | |  |

**Table 5.** Validated mRNA targets of DE miRNAs obtained from miRTarBase.

| **miRNA** | **Target** | **miRNA** | **Target** | **miRNA** | **Target** |
| --- | --- | --- | --- | --- | --- |
| hsa-miR-122-5p | AACS | hsa-miR-122-5p | AKT3 | hsa-miR-193a-3p | AURKA |
| hsa-miR-183-5p | AANAT | hsa-miR-122-5p |  | hsa-miR-24-3p | AURKB |
| hsa-miR-1-3p | ABCB1 | hsa-miR-152-3p | ALCAM | hsa-miR-122-5p | AXL |
| hsa-miR-24-3p | ABCB9 | hsa-miR-210-3p | ALDH5A1 | hsa-miR-34c-5p |  |
| hsa-miR-7-5p | ABCC1 | hsa-miR-122-5p | ALDOA | hsa-miR-196a-5p | BACH1 |
| hsa-miR-193a-3p | ABI2 | hsa-miR-193a-3p | ALKBH5 | hsa-miR-138-5p | BAG1 |
| hsa-miR-206 | ACTL6A | hsa-miR-204-5p | ALPL | hsa-miR-143-3p | BAG3 |
| hsa-miR-193a-3p | ACTN4 | hsa-miR-122-5p | ANK2 | hsa-miR-1-3p | BAG4 |
| hsa-miR-130a-3p | Acvr1 | hsa-miR-193a-3p | ANKFY1 | hsa-miR-127-3p | BAG5 |
| hsa-miR-130a-3p | Acvr1 | hsa-miR-204-5p | ANKRD13A | hsa-miR-182-5p | BARD1 |
| hsa-miR-24-3p | ACVR1B | hsa-miR-196a-5p | ANXA1 | hsa-miR-122-5p | BAX |
| hsa-miR-122-5p | ACVR1C | hsa-miR-122-5p | ANXA11 | hsa-miR-7-5p |  |
| hsa-miR-194-5p | ACVR2B | hsa-miR-1-3p | ANXA2 | hsa-miR-193a-3p | BAZ2A |
| hsa-miR-122-5p | ADAM10 | hsa-miR-206 |  | hsa-miR-24-3p | BCAR1 |
| hsa-miR-122-5p | ADAM17 | hsa-miR-204-5p | AP1S2 | hsa-miR-138-5p | BCL11A |
| hsa-miR-152-3p |  | hsa-miR-122-5p | AP3M2 | hsa-miR-126-3p | BCL2 |
| hsa-miR-126-3p | ADAM9 | hsa-miR-1-3p | API5 | hsa-miR-143-3p |  |
| hsa-miR-126-5p |  | hsa-miR-130a-3p | APP | hsa-miR-182-5p |  |
| hsa-miR-144-3p | Adamts1 | hsa-miR-144-3p |  | hsa-miR-184 |  |
| hsa-miR-1-3p | ADAR | hsa-miR-24-3p | ARHGAP19 | hsa-miR-204-5p |  |
| hsa-miR-182-5p | ADCY6 | hsa-miR-486-5p | ARHGAP5 | hsa-miR-34c-5p |  |
| hsa-miR-138-5p | ADGRA2 | hsa-miR-138-5p | ARHGEF3 | hsa-miR-7-5p |  |
| hsa-miR-126-3p | ADGRE5 | hsa-miR-193a-3p | ARMC1 | hsa-miR-122-5p | BCL2L1 |
| hsa-miR-126-3p | ADM | hsa-miR-335-5p | ARPC5L | hsa-miR-24-3p | BCL2L11 |
| hsa-miR-1-3p | AGO1 | hsa-miR-182-5p | ARRDC3 | hsa-miR-122-5p | BCL2L2 |
| hsa-miR-184 | AGO2 | hsa-miR-193a-3p | ASB3 | hsa-miR-129-2-3p |  |
| hsa-miR-24-3p | AGPAT2 | hsa-miR-1-3p | ASPH | hsa-miR-204-5p |  |
| hsa-miR-210-3p | AIFM3 | hsa-miR-182-5p | ATF1 | hsa-miR-335-5p |  |
| hsa-miR-210-3p | AIFM3 | hsa-miR-23b-3p | ATG12 | hsa-miR-335-5p |  |
| hsa-miR-193a-3p | AK2 | hsa-miR-152-3p | ATG14 | hsa-miR-127-3p | BCL6 |
| hsa-miR-183-5p | AKAP12 | hsa-miR-130a-3p | ATG2B | hsa-miR-146a-5p | BCLAF1 |
| hsa-miR-126-3p | AKT1 | hsa-miR-24-3p | ATG4A | hsa-miR-194-5p |  |
| hsa-miR-138-5p |  | hsa-miR-210-3p | ATG7 | hsa-miR-1-3p | BDNF |
| hsa-miR-143-3p |  | hsa-miR-122-5p | ATP1A2 | hsa-miR-182-5p |  |
| hsa-miR-184 |  | hsa-miR-140-3p | ATP6AP2 | hsa-miR-204-5p |  |
| hsa-miR-206 |  | hsa-miR-1-3p | ATP6V1B2 | hsa-miR-210-3p |  |
| hsa-miR-143-3p | AKT2 | hsa-miR-140-3p | ATP8A1 | hsa-miR-184 | BIN3 |
| hsa-miR-184 |  | hsa-miR-130a-3p | ATXN1 | hsa-miR-204-5p | BIRC2 |
| hsa-miR-335-5p | BIRC5 | hsa-miR-138-5p | CCND3 | hsa-miR-23b-3p | CHUK |
| hsa-miR-138-5p | BLCAP | hsa-miR-7-5p | CCNE1 | hsa-miR-193a-3p | CIAO1 |
| hsa-miR-34c-5p | BMF | hsa-miR-126-3p | CCNE2 | hsa-miR-486-5p | CIT |
| hsa-miR-183-5p | BMI1 | hsa-miR-34c-5p |  | hsa-miR-486-5p | CLDN10 |
| hsa-miR-194-5p |  | hsa-miR-122-5p | CCNG1 | hsa-miR-122-5p | CLIC4 |
| hsa-miR-210-3p | BNIP3 | hsa-miR-23b-3p | CCNG1 | hsa-miR-182-5p | CLOCK |
| hsa-miR-143-3p | BRAF | hsa-miR-129-2-3p | CCP110 | hsa-miR-193a-3p | CLPB |
| hsa-miR-146a-5p | BRCA1 | hsa-miR-152-3p | CD151 | hsa-miR-574-3p | CLTC |
| hsa-miR-24-3p |  | hsa-miR-138-5p | CD274 | hsa-miR-1-3p | CNN3 |
| hsa-miR-335-5p |  | hsa-miR-152-3p |  | hsa-miR-146a-5p | CNOT6L |
| hsa-miR-146a-5p | BRCA2 | hsa-miR-140-3p | CD38 | hsa-miR-143-3p | COL1A1 |
| hsa-miR-210-3p | BTK | hsa-miR-146a-5p | CD40LG | hsa-miR-143-3p | COL3A1 |
| hsa-miR-183-5p | BTRC | hsa-miR-143-3p | CD44 | hsa-miR-140-3p | COL4A1 |
| hsa-miR-193a-3p | BUB1 | hsa-miR-146a-5p | CD80 | hsa-miR-210-3p | COL4A2 |
| hsa-miR-193a-3p | C1QBP | hsa-miR-204-5p | CDC42 | hsa-miR-24-3p | COPS5 |
| hsa-miR-193a-3p | C5orf22 | hsa-miR-138-5p | CDH1 | hsa-miR-146a-5p | COPS8 |
| hsa-miR-193a-3p | C6orf106 | hsa-miR-204-5p |  | hsa-miR-193a-3p | COQ7 |
| hsa-miR-193a-3p | C6orf47 | hsa-miR-194-5p | CDH2 | hsa-miR-24-3p | CORO1A |
| hsa-miR-23b-3p | CA2 | hsa-miR-24-3p | CDK1 | hsa-miR-1-3p | COX1 |
| hsa-miR-193a-3p | CACFD1 | hsa-miR-193a-3p | CDK12 | hsa-miR-146a-5p | COX2 |
| hsa-miR-126-3p | CADM1 | hsa-miR-122-5p | CDK4 | hsa-miR-210-3p | CPEB2 |
| hsa-miR-182-5p |  | hsa-miR-1-3p |  | hsa-miR-146a-5p | CPM |
| hsa-miR-1-3p | CALM3 | hsa-miR-206 |  | hsa-miR-193a-3p | CPSF2 |
| hsa-miR-1-3p | CAND1 | hsa-miR-24-3p |  | hsa-miR-122-5p | CREB1 |
| hsa-miR-146a-5p | CARD10 | hsa-miR-34c-5p |  | hsa-miR-182-5p |  |
| hsa-miR-24-3p |  | hsa-miR-486-5p |  | hsa-miR-204-5p | CREB1 |
| hsa-miR-138-5p | CASP3 | hsa-miR-129-2-3p | CDK6 | hsa-miR-182-5p | CREB5 |
| hsa-miR-146a-5p | CASP7 | hsa-miR-34c-5p |  | hsa-miR-204-5p |  |
| hsa-miR-210-3p | CASP8AP2 | hsa-miR-146a-5p | CDKN1A | hsa-miR-126-3p | CRK |
| hsa-miR-193a-3p | CCDC8 | hsa-miR-182-5p |  | hsa-miR-126-5p |  |
| hsa-miR-152-3p | CCKBR | hsa-miR-196a-5p |  | hsa-miR-126-3p | CRKL |
| hsa-miR-1-3p | CCL2 | hsa-miR-182-5p | CDKN1B | hsa-miR-335-5p | CRKL |
| hsa-miR-206 |  | hsa-miR-190a-5p |  | hsa-miR-130a-3p | CSF1 |
| hsa-miR-146a-5p | CCL5 | hsa-miR-194-5p |  | hsa-miR-152-3p | CSF1 |
| hsa-miR-146a-5p | CCNA2 | hsa-miR-196a-5p |  | hsa-miR-193a-3p | CTC1 |
| hsa-miR-24-3p | CCNA2 | hsa-miR-24-3p |  | hsa-miR-122-5p | CTDNEP1 |
| hsa-miR-138-5p | CCND1  CCND1 | hsa-miR-24-3p | CDKN2A | hsa-miR-143-3p | CTGF |
| hsa-miR-1-3p |  | hsa-miR-146a-5p | CDKN3 | hsa-miR-574-3p | CUL2 |
| hsa-miR-146a-5p |  | hsa-miR-204-5p | CDX2 | hsa-miR-7-5p | CUL5 |
| hsa-miR-152-3p |  | hsa-miR-1-3p | CEBPA | hsa-miR-126-3p | CXCL12 |
| hsa-miR-193a-3p |  | hsa-miR-193a-3p | CEP89 | hsa-miR-126-5p |  |
| hsa-miR-206 |  | hsa-miR-146a-5p | CFH | hsa-miR-1-3p |  |
| hsa-miR-24-3p |  | hsa-miR-144-3p | CFTR | hsa-miR-146a-5p |  |
| hsa-miR-34c-5p |  | hsa-miR-194-5p | CHD1 | hsa-miR-146a-5p | CXCL8 |
| hsa-miR-146a-5p | CCND2 | hsa-miR-24-3p | CHEK1 | hsa-miR-126-3p | CXCR4 |
| hsa-miR-182-5p |  | hsa-miR-182-5p | CHEK2 | hsa-miR-146a-5p |  |
| hsa-miR-206 |  | hsa-miR-182-5p | CHL1 | hsa-miR-204-5p |  |
| hsa-miR-204-5p | CYBB | hsa-miR-24-3p | CYP11B2 | hsa-miR-335-5p |  |
| hsa-miR-143-3p | DDX6 | hsa-miR-183-5p | DKK3 | hsa-miR-126-5p | CYLD |
| hsa-miR-24-3p | DEDD | hsa-miR-130a-3p | DLL4 | hsa-miR-182-5p |  |
| hsa-miR-24-3p | DHFR | hsa-miR-193a-3p | DNAJB9 | hsa-miR-143-3p | CYP2C9 |
| hsa-miR-24-3p | DHFRP1 | hsa-miR-24-3p | DND1 | hsa-miR-122-5p | CYP7A1 |
| hsa-miR-130a-3p | DICER1 | hsa-miR-126-3p | DNMT1 | hsa-miR-138-5p | CYTOR |
| hsa-miR-210-3p | DIMT1 | hsa-miR-152-3p |  | hsa-miR-335-5p | DAAM2 |
| hsa-miR-152-3p | DKK1 | hsa-miR-143-3p | DNMT3A | hsa-miR-193a-3p | DCAF7 |
| hsa-miR-335-5p |  | hsa-miR-194-5p |  | hsa-miR-193a-3p | DCTN5 |
| hsa-miR-486-5p | DOCK3 | hsa-miR-122-5p | EGFR | hsa-miR-193a-3p | DDAH1 |
| hsa-miR-122-5p | DSTYK | hsa-miR-133a-5p |  | hsa-miR-210-3p |  |
| hsa-miR-143-3p | DTNB | hsa-miR-146a-5p |  | hsa-miR-130a-3p | ESR1 |
| hsa-miR-146a-5p | DUSP1 | hsa-miR-574-3p |  | hsa-miR-206 |  |
| hsa-miR-122-5p | DUSP2 | hsa-miR-7-5p |  | hsa-miR-1-3p | ETS1 |
| hsa-miR-204-5p | DVL3 | hsa-miR-122-5p | EGLN3 | hsa-miR-144-3p |  |
| hsa-miR-193a-3p | DYRK2 | hsa-miR-183-5p | EGR1 | hsa-miR-23b-3p |  |
| hsa-miR-24-3p |  | hsa-miR-210-3p | EHD2 | hsa-miR-126-3p | EZH2 |
| hsa-miR-193a-3p | E2F1 | hsa-miR-138-5p | EID1 | hsa-miR-138-5p |  |
| hsa-miR-24-3p | E2F2 | hsa-miR-24-3p | EIF2S3 | hsa-miR-138-5p |  |
| hsa-miR-210-3p | E2F3 | hsa-miR-138-5p | EIF4EBP1 | hsa-miR-144-3p |  |
| hsa-miR-34c-5p |  | hsa-miR-146a-5p | ELAVL1 | hsa-miR-183-5p | EZR |
| hsa-miR-193a-3p | E2F6 | hsa-miR-193a-3p | ELMO2 | hsa-miR-184 |  |
| hsa-miR-193a-3p | EBAG9 | hsa-miR-196a-5p | ELOVL1 | hsa-miR-204-5p |  |
| hsa-miR-204-5p | EDEM1 | hsa-miR-204-5p | ELOVL6 | hsa-miR-1-3p | FABP3 |
| hsa-miR-1-3p | EDN1 | hsa-miR-122-5p | ENTPD4 | hsa-miR-146a-5p | FADD |
| hsa-miR-138-5p | EED | hsa-miR-574-3p | EP300 | hsa-miR-146a-5p | FAF1 |
| hsa-miR-210-3p | EFNA3 | hsa-miR-335-5p | EPN2 | hsa-miR-24-3p |  |
| hsa-miR-204-5p | EFNB2 | hsa-miR-193a-3p | ERBB2 | hsa-miR-122-5p | FAM117B |
| hsa-miR-126-3p | EGFL7 | hsa-miR-146a-5p | ERBB4 | hsa-miR-193a-3p | FAM221B |
| hsa-miR-143-3p | FAM83F | hsa-miR-193a-3p |  | hsa-miR-423-5p | FAM3A |
| hsa-miR-146a-5p | FANCM | hsa-miR-144-3p | FGB | hsa-miR-210-3p | Fgfrl1 |
| hsa-miR-146a-5p | FAS | hsa-miR-24-3p | FGF11 | hsa-miR-144-3p | FGG |
| hsa-miR-1-3p | FASN | hsa-miR-152-3p | FGF2 | hsa-miR-143-3p | FHIT |
| hsa-miR-486-5p | FBN1 | hsa-miR-182-5p | FGF9 | hsa-miR-182-5p | FLOT1 |
| hsa-miR-182-5p | FBXW7 | hsa-miR-152-3p | FGFR3 | hsa-miR-335-5p | FMN2 |
| hsa-miR-24-3p |  | hsa-miR-24-3p |  | hsa-miR-335-5p | FMNL3 |
| hsa-miR-24-3p | FEN1 | hsa-miR-210-3p | FGFRL1 | hsa-miR-138-5p | FERMT2 |
| hsa-miR-144-3p | FGA |  |  |  |  |

**Table 6.** Gene ontology terms for each miRNA. Not all miRNAs have associated GO terms

| **miRNA** | **Gene Ontology Terms** | | | | | | | |
| --- | --- | --- | --- | --- | --- | --- | --- | --- |
| miR-194b-5p | negative regulation of interleukin-10 production | | | | | | | |
| miR-7 | negative regulation of amyloid-beta clearance | | negative regulation of insulin receptor signaling pathway | | | | negative regulation of sprouting angiogenesis | |
| miR-499 | positive regulation of blood vessel endothelial cell migration | | positive regulation of vascular associated smooth muscle cell proliferation | | positive regulation of vascular associated smooth muscle cell migration | | positive regulation of vascular endothelial cell proliferation | |
| miR-193a | cellular response to DNA damage stimulus | | negative regulation of cell migration involved in sprouting angiogenesis | | negative regulation of blood vessel endothelial cell proliferation involved in sprouting angiogenesis | | negative regulation of G1/S transition of mitotic cell cycle | |
| miR-144 | negative regulation of glycoprotein biosynthetic process | negative regulation of epithelial to mesenchymal transition | positive regulation of mitochondrion organization | positive regulation of cell adhesion molecule production | positive regulation of cholesterol storage | positive regulation of high-density lipoprotein particle clearance | | positive regulation of interleukin-1 beta production |
| miR-144 | positive regulation of interleukin-6 production | positive regulation of tumor necrosis factor production | cholesterol homeostasis | negative regulation of amyloid precursor protein biosynthetic process | positive regulation of inflammatory response | regulation of high-density lipoprotein particle assembly | negative regulation of cholesterol efflux | negative regulation of reverse cholesterol transport |
| miR-183 | negative regulation of cell-substrate adhesion | | positive regulation of phagocytosis | | transforming growth factor beta receptor signaling pathway | | | |
| miR-196a | negative regulation of lamellipodium assembly | cellular response to vascular endothelial growth factor stimulus | negative regulation of cell migration involved in sprouting angiogenesis | negative regulation of histone deacetylase activity | negative regulation of epithelial cell apoptotic process | negative regulation of vascular associated smooth muscle cell proliferation | negative regulation of vascular associated smooth muscle cell migration | negative regulation of vascular associated smooth muscle cell dedifferentiation |

**Table 6.** Gene ontology terms for each miRNA. Not all miRNAs have associated GO terms (Continued)

| **miRNA** | **Gene Ontology Terms** | | | | | | | | | | | | | | |  |
| --- | --- | --- | --- | --- | --- | --- | --- | --- | --- | --- | --- | --- | --- | --- | --- | --- |
| miR-182 | positive regulation of cytokine production | | positive regulation of cell migration | | cholesterol homeostasis | | positive regulation of cholesterol biosynthetic process | | positive regulation of fatty acid biosynthetic process | | positive regulation of lipoprotein lipase activity | | cellular response to cholesterol | | positive regulation of NIK/NF-kappaB signaling | |
|  | negative regulation of stress fiber assembly | positive regulation of protein kinase B signaling | | negative regulation of response to cytokine stimulus | | negative regulation of protein K63-linked ubiquitination | | negative regulation of sprouting angiogenesis | | | | negative regulation of vascular associated smooth muscle cell apoptotic process | | | |  |
| miR-138 | negative regulation of cell adhesion | negative regulation of cell population proliferation | | negative regulation of cell migration | | negative regulation of NF-kappaB transcription factor activity | | negative regulation of osteoblast proliferation | | negative regulation of osteoblast differentiation | | negative regulation of inflammatory response | | negative regulation of nitric-oxide synthase activity | |  |
| miR-126-5p | negative regulation of cell migration | negative regulation of proteolysis | | positive regulation of Notch signaling pathway | | cellular response to laminar fluid shear stress | | positive regulation of cell migration involved in sprouting angiogenesis | | positive regulation of blood vessel endothelial cell proliferation involved in sprouting angiogenesis | | positive regulation of vascular endothelial cell proliferation | | positive regulation of vascular endothelial cell proliferation | |  |

**Table 6.** Gene ontology terms for each miRNA. Not all miRNAs have associated GO terms (Continued)

| **miRNA** | **Gene Ontology Terms** | | | | | | | | |
| --- | --- | --- | --- | --- | --- | --- | --- | --- | --- |
| miR-130a | negative regulation of tumor necrosis factor production | NIK/NF-kappaB signaling | negative regulation of macrophage activation | positive regulation of angiogenesis | cellular response to transforming growth factor beta stimulus | cellular response to virus | regulation of viral life cycle | positive regulation of vascular associated smooth muscle cell proliferation | |
|  | positive regulation of blood vessel endothelial cell proliferation involved in sprouting angiogenesis | | | negative regulation of sprouting angiogenesis | | | | | |
| miR-23b | negative regulation of signaling receptor activity | negative regulation of interleukin-11 production | cellular response to vascular endothelial growth factor stimulus | positive regulation of vascular permeability | positive regulation of cardiac muscle cell proliferation | cell growth involved in cardiac muscle cell development | positive regulation of ERK1 and ERK2 cascade | positive regulation of cell migration involved in sprouting angiogenesis | |
|  | negative regulation of intracellular signal transduction | negative regulation of matrix metallopeptidase secretion | negative regulation of membrane permeability | negative regulation of chemokine (C-X-C motif) ligand 2 production | negative regulation of endothelial cell proliferation | negative regulation of angiogenesis | regulation of toll-like receptor signaling pathway | negative regulation of toll-like receptor 4 signaling pathway | NIK/NF-kappaB signaling |
| miR-146a | negative regulation of cellular extravasation | negative regulation of cholesterol storage | negative regulation of interleukin-6 production | negative regulation of interleukin-8 production | negative regulation of dephosphorylation | positive regulation of apoptotic process | negative regulation of inflammatory response | negative regulation of protein kinase B signaling | |

**Table 6.** Gene ontology terms for each miRNA. Continued

| **miRNA** | **Gene Ontology Terms** | | | | | | | | | | | | |
| --- | --- | --- | --- | --- | --- | --- | --- | --- | --- | --- | --- | --- | --- |
| miR-152 | negative regulation of tumor necrosis factor-mediated signaling pathway | | negative regulation of blood vessel endothelial cell migration | | | negative regulation of fibroblast growth factor production | | | negative regulation of metalloendopeptidase activity | | | negative regulation of vascular endothelial cell proliferation | |
| miR-143-3p | regulation of smooth muscle contraction | | | | | | | | | | | | |
|  | negative regulation of smooth muscle cell proliferation | negative regulation of angiogenesis | | actin cytoskeleton organization | activation of protein kinase B activity | | angiotensin-activated signaling pathway | positive regulation of blood vessel endothelial cell migration | | establishment or maintenance of cell type involved in phenotypic switching | positive regulation of angiogenesis | | negative regulation of glucose import |
| miR-1 | negative regulation of xenobiotic detoxification by transmembrane export across the plasma membrane | | | positive regulation of protein kinase B signaling | aorta smooth muscle tissue morphogenesis | | regulation of phenotypic switching | positive regulation of vascular associated smooth muscle cell migration | | | positive regulation of pulmonary blood vessel remodeling | | |
|  | negative regulation of insulin-like growth factor receptor signaling pathway | | | positive regulation of cell fate commitment | positive regulation of heart rate | | negative regulation of cardiac muscle hypertrophy | positive regulation of myotube differentiation | | regulation of release of sequestered calcium ion into cytosol by sarcoplasmic reticulum | | | regulation of cardiac muscle contraction by regulation of the release of sequestered calcium ion |

**Table 6.** Gene ontology terms for each miRNA. Continued

| **miRNA** | **Gene Ontology Terms** | | | | | | | | |
| --- | --- | --- | --- | --- | --- | --- | --- | --- | --- |
| miR-1 | negative regulation of canonical Wnt signaling pathway | | negative regulation of endothelial cell differentiation | negative regulation of cardiac muscle cell proliferation | positive regulation of sarcomere organization | regulation of ventricular cardiac muscle cell membrane depolarization | ventricular septum morphogenesis | positive regulation of cardiac muscle contraction | cell migration involved in coronary vasculogenesis |
|  | positive regulation of vascular associated smooth muscle cell apoptotic process | negative regulation of delayed rectifier potassium channel activity | positive regulation of sprouting angiogenesis | positive regulation of voltage-gated potassium channel activity involved in ventricular cardiac muscle cell action potential repolarization | | negative regulation of cardiac conduction | negative regulation of vascular associated smooth muscle cell proliferation | positive regulation of calcium ion transmembrane transport via high voltage-gated calcium channel | negative regulation of membrane repolarization during cardiac muscle cell action potential |
| miR-204 | negative regulation of interleukin-6 production | positive regulation of mesoderm formation | negative regulation of calcium ion export across plasma membrane | positive regulation of cardiac vascular smooth muscle cell differentiation | positive regulation of cardiac muscle cell differentiation | negative regulation of myoblast proliferation | positive regulation of skeletal muscle cell differentiation | positive regulation of ryanodine-sensitive calcium-release channel activity by adrenergic receptor signaling pathway involved in positive regulation of cardiac muscle contraction | |
|  | negative regulation of blood vessel endothelial cell migration | negative regulation of interleukin-8 production | negative regulation of tumor necrosis factor production | negative regulation of NIK/NF-kappaB signaling | negative regulation of cell population proliferation | negative regulation of cell migration | negative regulation of prostaglandin biosynthetic process | negative regulation of interleukin-1 beta production | positive regulation of apoptotic process |

**Table 6.** Gene ontology terms for each miRNA. Continued

| **miRNA** | **Gene Ontology Terms** | | | | | | | | |
| --- | --- | --- | --- | --- | --- | --- | --- | --- | --- |
| miR-126-3p | negative regulation of cell migration | negative regulation of inflammatory response | | positive regulation of cardiac muscle cell proliferation | | negative regulation of cardiac muscle myoblast proliferation | | positive regulation of cardiac muscle cell differentiation | |
|  | positive regulation of ERK1 and ERK2 cascade | negative regulation of proteolysis | positive regulation of phosphatidylinositol 3-kinase signaling | positive regulation of MAPK cascade | positive regulation of blood vessel endothelial cell migration | positive regulation of angiogenesis | negative regulation of inflammatory response | positive regulation of inflammatory response | positive regulation of protein kinase B signaling |
| miR-210 | negative regulation of neuron projection development | positive regulation of cell migration involved in sprouting angiogenesis | | positive regulation of sprouting angiogenesis | positive regulation of vasculature development | negative regulation of vascular endothelial cell proliferation | negative regulation of endothelial cell apoptotic process | positive regulation of blood vessel endothelial cell proliferation involved in sprouting angiogenesis | |
|  | positive regulation of glucose catabolic process to lactate via pyruvate | positive regulation of cell migration | tube formation | positive regulation of blood vessel endothelial cell migration | positive regulation of osteoblast differentiation | positive regulation of angiogenesis | hypoxia-inducible factor-1alpha signaling pathway | regulation of cellular response to hypoxia | negative regulation of mitochondrial electron transport, NADH to ubiquinone |

**Table 6.** Gene ontology terms for each miRNA. Continued

| miR-24-3p | negative regulation of interferon-gamma production | negative regulation of aconitate hydratase activity | positive regulation of iron ion import across plasma membrane | | negative regulation of apoptotic signaling pathway | positive regulation of apoptotic signaling pathway | | negative regulation of vascular associated smooth muscle cell apoptotic process | |
| --- | --- | --- | --- | --- | --- | --- | --- | --- | --- |
|  | positive regulation of reactive oxygen species biosynthetic process | negative regulation of amyloid-beta formation | negative regulation of cardiac muscle cell apoptotic process | negative regulation of tumor necrosis factor-mediated signaling pathway | negative regulation of angiogenesis | negative regulation of blood vessel endothelial cell migration | cell growth involved in cardiac muscle cell development | positive regulation of ERK1 and ERK2 cascade | negative regulation of protein serine/threonine kinase activity |
| miR-335 | negative regulation of cell population proliferation | | | | negative regulation of cell migration | | | | |
| miR-34c | negative regulation of protein kinase B signaling | | | positive regulation of cardiac muscle hypertrophy in response to stress | | | negative regulation of sprouting angiogenesis | | |

**Table 7.** Representative gene ontology terms shared between at least two DE miRNAs

| **miRNA ID** | **Age(months)** | **Gene Ontology Term** |
| --- | --- | --- |
| miR-138-5p  miR-126-5p  miR-204-5p  miR-126-3p  miR-335-5p | 3, 6  3  6  6, 9  9 | Negative regulation of cell migration |
| miR-144-3p  miR-182-5p | 3  3 | Cholesterol homeostasis |
| miR-138-5p  miR-146a-5p  miR-204  miR-126-3p | 3, 6  3  6  6, 9 | Negative regulation of inflammatory responses |
| miR-146a-5p  miR-204-5p | 3  6 | Negative regulation of interleukin 6 and interleukin 8 |
| miR-130a-3p  miR-204-5p | 3, 6  6 | Negative regulation of tumor necrosis factor production |
| miR-182-5p  miR-1-3p | 3  6 | Negative regulation of vascular associated smooth muscle cell proliferation |
| miR-182-5p  miR-130a-3p | 3  3, 6 | Positive regulation of NIK/NF Kappa beta signaling |
| miR-146a-5p  miR-204 | 3  6 | Negative regulation of NIK/NF Kappa beta signaling |
| miR-138-5p  miR-146a-5p | 3, 6  3 | Negative regulation of cell adhesion |
| miR-138-5p  miR-204-5p  miR-335 | 3, 6  6  9 | Negative regulation of cell proliferation |
| miR-23b-3p  miR-196a-5p | 3  3 | Cellular response to vascular endothelial growth factor stimulus |
| miR-23b-3p  miR-204-5p | 3  6 | Positive regulation of cardiac muscle cell proliferation |
| miR-23b-3p  miR-24-3p | 3  6 | Cell growth involved in cardiac muscle cell development |
| miR-23b-3p  miR-126-3p  miR-24-3p | 3  6, 9  6 | Positive regulation of ERK1 and ERK2 cascade |
| miR-23b-3p  miR-126-5p  miR-146a-5p  miR-126-3p | 3  3  3  6, 9 | Positive regulation of cell migration involved in sprouting angiogenesis  Positive regulation of blood vessel endothelial cell proliferation involved in sprouting angiogenesis |
| miR-23b-3p  miR-7-5p  miR-138-5p  miR-34c-5p | 3  3  3, 6  9 | Negative regulation of sprouting angiogenesis |
| miR-499a-3p  miR-143-3p  miR-126-3p  miR-210-3p | 3  6  3  6 | Positive regulation of blood vessel endothelial cell migration |
| miR-499a-3p  miR-130a-3p | 3  3, 6 | Positive regulation of vascular associated smooth muscle cell proliferation |
| miR-499a-3p  miR-143-3p | 3  6 | Positive regulation of vascular associated smooth muscle cell migration |
| miR-499-3p  miR-126-5p  miR-130a-3p | 3  3  3, 6 | Positive regulation of vascular endothelial cell proliferation |
| miR-193a-3p  miR-196a-5p  miR-146a-5p | 3  3  3 | Negative regulation of cell migration involved in sprouting angiogenesis |
| miR-193a-3p  miR-24-3p | 3  6 | Negative regulation of blood vessel endothelial cell proliferation involving sprouting angiogenesis |
| miR-146a-5p  miR-152-3p  miR-24-3p | 3  3  6 | Negative regulation of matrix metallopeptidase secretion and activity |
| miR-146a-5p  miR-143-3p  miR-24-3p | 3  6  6 | Negative regulation of angiogenesis |
| miR-130a-3p  miR-143-3p  miR-126-3p  miR-210-3p | 3, 6  6  3, 9  6 | Positive regulation of angiogenesis |
| miR-152-3p  miR-204-5p  miR-24-3p | 3  6  6 | Negative regulation of blood vessel endothelial cell migration |

**Table 8.** Differential expression of miRNAs in WMS-FH (n =15) relative to WMS-N (n=15) in a new cohort of animals via RT-qPCR.

| **miRNA** | **Fold change** | | | **Linear regression model p-value** | | |
| --- | --- | --- | --- | --- | --- | --- |
|  | **Month 3** | **Month 6** | **Month 9** | **Month**  **3** | **Month**  **6** | **Month**  **9** |
| **miR-194-5p**  ↑in WMS-FH | 1.42 | 1.58 | 3.31 | Weight: 0.722  Hemolysis: 0.016  Sex: <0.001  Group: 0.425 | 0.019  0.007  0.970  **0.007** | 0.126  0.126  **0.036**  **0.042** |
| **miR-206**  ↑in WMS-FH | - | 1.82 | - | Weight: -  Hemolysis: -  Sex: -  Group: - | 0.023  0.204  0.354  **0.032** | -  -  -  - |
| **miR-1**  ↓ in WMS-FH | - | 0.84 | - | Weight: -  Hemolysis: -  Sex: -  Group: - | 0.774  0.060  0.340  0.660 | -  -  -  - |
| **miR-138-5p**  ↓ in WMS-FH | 0.70 | 0.45 | - | Weight: 0.082  Hemolysis: 0.001  Sex: 0.008  Group: 0.281 | 0.368  0.004  0.337  **0.005** | -  -  -  - |
| **miR-126-3p**  ↓ in WMS-FH | - | 0.71 | - | Weight: -  Hemolysis: -  Sex: -  Group: - | 0.104  0.003  0.494  **0.021** | -  -  -  - |

-: Not tested.


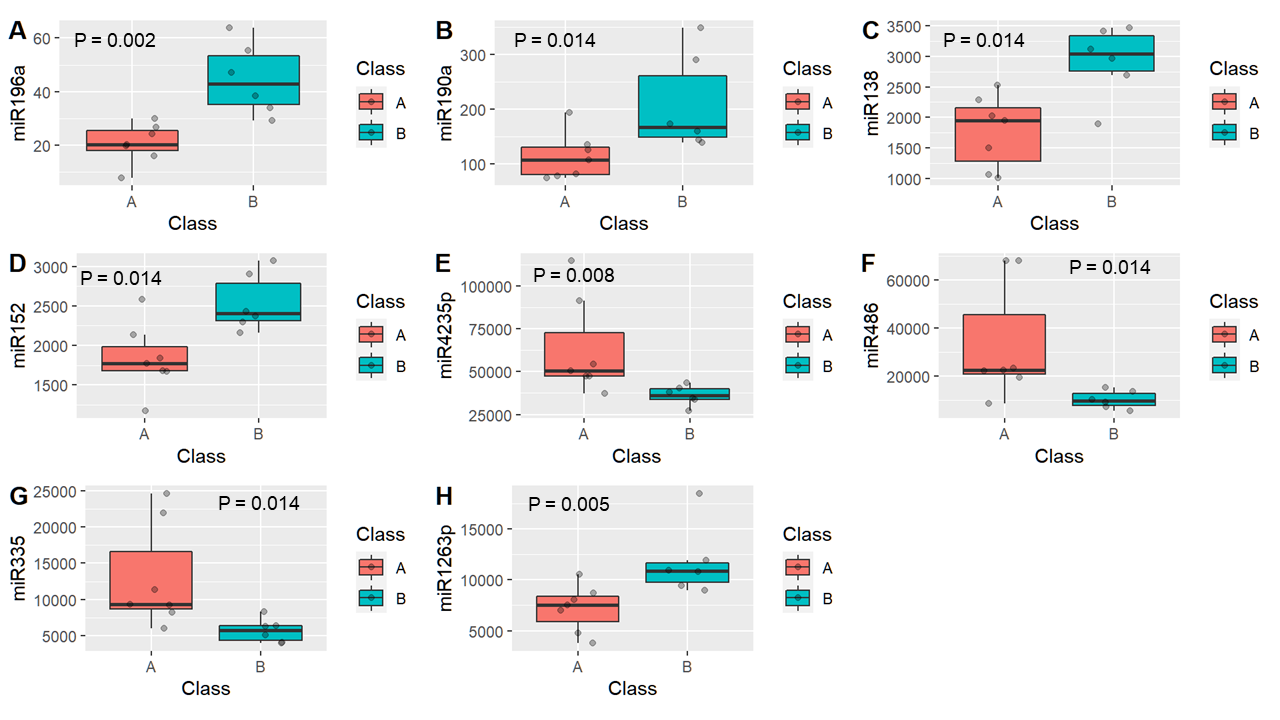


**Figure 1.** Boxplot of normalized reads for miRNA with discriminatory power between WMS animals with coronary artery disease (Class A) and healthy (Class B).

**Table 9.** Global terms grouping Gene Ontology terms obtained for DE miRNAs

| **Gene Ontology Term** | **Global term** | **Month** |
| --- | --- | --- |
| regulation of reverse cholesterol transport | Cholesterol homeostasis | Upregulated at Month 3 |
| cholesterol homeostasis |  |  |
| regulation of cholesterol efflux |  |  |
| regulation of cholesterol storage |  |  |
| regulation of high-density lipoprotein particle assembly |  |  |
| regulation of high-density lipoprotein particle clearance |  |  |
| regulation of blood vessel endothelial cell migration | Endothelial cells proliferation/migration |  |
| regulation of blood vessel endothelial cell proliferation involved in sprouting angiogenesis |  |  |
| regulation of vascular endothelial cell proliferation |  |  |
| regulation of tumor necrosis factor production | Inflammatory processes |  |
| regulation of cell adhesion molecule production |  |  |
| regulation of inflammatory response |  |  |
| regulation of interleukin-1 beta production | Interleukin production |  |
| regulation of interleukin-6 production |  |  |
| regulation of interleukin-10 production |  |  |
| regulation of amyloid-beta clearance | Other |  |
| regulation of insulin receptor signaling pathway |  |  |
| regulation of mitochondrion organization |  |  |
| regulation of epithelial to mesenchymal transition |  |  |
| regulation of G1/S transition of mitotic cell cycle |  |  |
| regulation of glycoprotein biosynthetic process |  |  |
| regulation of amyloid precursor protein biosynthetic process |  |  |

**Table 9.** Global terms grouping Gene Ontology terms obtained for DE miRNAs (Continued)

| **Gene Ontology Term** | **Global term** | **Month** |
| --- | --- | --- |
| regulation of cell migration involved in sprouting angiogenesis | Sprouting and angiogenesis | Upregulated at Month 3 |
| regulation of sprouting angiogenesis |  |  |
| regulation of vascular associated smooth muscle cell proliferation | VSMC proliferation/migration |  |
| regulation of vascular associated smooth muscle cell migration |  |  |
| cellular response to cholesterol | Cholesterol homeostasis | Downregulated at Month 3 |
| regulation of cholesterol biosynthetic process |  |  |
| regulation of cholesterol storage |  |  |
| regulation of fatty acid biosynthetic process |  |  |
| cellular response to oxidised low-density lipoprotein particle stimulus |  |  |
| regulation of lipoprotein lipase activity |  |  |
| cholesterol homeostasis |  |  |
| regulation of vascular endothelial cell proliferation | Endothelial cells proliferation/migration |  |
| regulation of blood vessel endothelial cell proliferation involved in sprouting angiogenesis |  |  |
| regulation of endothelial cell proliferation |  |  |
| regulation of blood vessel endothelial cell migration |  |  |
| regulation of histone deacetylase activity | Epigenetic regulation |  |
| NIK/NF-kappaB signaling | Inflammatory processes |  |
| regulation of protein kinase B signaling |  |  |

**Table 9.** Global terms grouping Gene Ontology terms obtained for DE miRNAs (Continued)

| **Gene Ontology Term** | **Global term** | **Month** |
| --- | --- | --- |
| regulation of inflammatory response | Inflammatory processes | Downregulated at Month 3 |
| cellular response to vascular endothelial growth factor stimulus |  |  |
| regulation of cellular extravasation |  |  |
| regulation of response to cytokine stimulus |  |  |
| regulation of cellular senescence |  |  |
| regulation of chemokine (C-X-C motif) ligand 2 production |  |  |
| regulation of tumor necrosis factor production |  |  |
| regulation of vascular endothelial growth factor production |  |  |
| regulation of cytokine production |  |  |
| regulation of cytokine production involved in inflammatory response |  |  |
| regulation of osteoblast differentiation |  |  |
| regulation of apoptotic process in bone marrow cell |  |  |
| regulation of epithelial cell apoptotic process |  |  |
| regulation of tyrosine phosphorylation of STAT protein |  |  |
| regulation of ERK1 and ERK2 cascade |  |  |
| regulation of Notch signaling pathway |  |  |
| regulation of apoptotic process |  |  |
| regulation of toll-like receptor 4 signaling pathway |  |  |
| regulation of tumor necrosis factor-mediated signaling pathway |  |  |
| regulation of leukocyte adhesion to vascular endothelial cell |  |  |
| regulation of macrophage activation |  |  |
| cellular response to cytokine stimulus |  |  |
| regulation of cell adhesion |  |  |
| cellular response to lipopolysaccharide |  |  |
| cellular response to transforming growth factor beta stimulus |  |  |
| regulation of cell adhesion molecule production |  |  |

**Table 9.** Global terms grouping Gene Ontology terms obtained for DE miRNAs (Continued)

| **Gene Ontology Term** | **Global term** | **Month** |
| --- | --- | --- |
| regulation of nitric-oxide synthase activity | Inflammatory processes | Downregulated at Month 3 |
| regulation of matrix metallopeptidase secretion |  |  |
| interleukin-1-mediated signaling pathway |  |  |
| regulation of metalloendopeptidase activity |  |  |
| regulation of toll-like receptor signaling pathway |  |  |
| transforming growth factor beta receptor signaling pathway |  |  |
| regulation of NF-kappaB transcription factor activity |  |  |
| regulation of NIK/NF-kappaB signaling |  |  |
| regulation of interleukin-11 production |  |  |
| regulation of interleukin-6 production |  |  |
| regulation of interleukin-8 production |  |  |
| regulation of cell migration |  |  |
| cellular response to virus |  |  |
| regulation of phagocytosis |  |  |
| cellular response to laminar fluid shear stress |  |  |
| regulation of dephosphorylation |  |  |
| regulation of cell population proliferation |  |  |
| cell growth involved in cardiac muscle cell development |  |  |
| regulation of fibroblast growth factor production |  |  |
| cellular response to glucose stimulus |  |  |
| regulation of fibroblast growth factor receptor signaling pathway |  |  |
| regulation of glial cell proliferation |  |  |
| regulation of osteoblast proliferation |  |  |
| regulation of protein K63-linked ubiquitination |  |  |

**Table 9.** Global terms grouping Gene Ontology terms obtained for DE miRNAs (Continued)

| **Gene Ontology Term** | **Global term** | **Month** |
| --- | --- | --- |
| regulation of proteolysis | Inflammatory processes | Downregulated at Month 3 |
| regulation of signaling receptor activity |  |  |
| regulation of intracellular signal transduction |  |  |
| regulation of lamellipodium assembly |  |  |
| regulation of cell-substrate adhesion |  |  |
| regulation of vascular permeability |  |  |
| regulation of cardiac muscle cell proliferation |  |  |
| cellular response to amyloid-beta |  |  |
| cellular response to hypoxia |  |  |
| regulation of stem cell differentiation |  |  |
| regulation of viral life cycle |  |  |
| regulation of membrane permeability |  |  |
| plasma membrane raft assembly |  |  |
| regulation of neuron differentiation |  |  |
| regulation of cell migration involved in sprouting angiogenesis | Sprouting and angiogenesis |  |
| regulation of angiogenesis |  |  |
| regulation of sprouting angiogenesis |  |  |
| regulation of vascular associated smooth muscle cell proliferation | VSMC proliferation/migration |  |
| regulation of vascular associated smooth muscle cell dedifferentiation |  |  |
| regulation of vascular associated smooth muscle cell apoptotic process |  |  |
| regulation of vascular associated smooth muscle cell migration |  |  |

**Table 9.** Global terms grouping Gene Ontology terms obtained for DE miRNAs (Continued)

| **Gene Ontology Term** | **Global term** | **Month** |
| --- | --- | --- |
| positive regulation of blood vessel endothelial cell migration | Endothelial cells proliferation/migration | Upregulated at Month 6 |
| negative regulation of blood vessel endothelial cell migration |  |  |
| negative regulation of endothelial cell differentiation |  |  |
| angiotensin-activated signaling pathway | Inflammatory processes |  |
| negative regulation of canonical Wnt signaling pathway |  |  |
| negative regulation of fibroblast growth factor receptor signaling pathway |  |  |
| negative regulation of inflammatory response |  |  |
| negative regulation of insulin-like growth factor receptor signaling pathway |  |  |
| negative regulation of NIK/NF-kappaB signaling |  |  |
| negative regulation of tumor necrosis factor production |  |  |
| negative regulation of interleukin-1 beta production | Interleukin production |  |
| negative regulation of interleukin-10 production |  |  |
| negative regulation of interleukin-6 production |  |  |
| negative regulation of interleukin-8 production |  |  |
| positive regulation of ryanodine-sensitive calcium-release channel activity by adrenergic receptor signaling pathway involved in positive regulation of cardiac muscle contraction | Muscle contraction |  |
| regulation of smooth muscle contraction |  |  |
| actin cytoskeleton organization |  |  |
| positive regulation of cardiac muscle contraction |  |  |
| positive regulation of sarcomere organization |  |  |
| negative regulation of membrane repolarization during cardiac muscle cell action potential |  |  |
| regulation of cardiac muscle contraction by regulation of the release of sequestered calcium ion |  |  |
| regulation of ventricular cardiac muscle cell membrane depolarization |  |  |

**Table 9.** Global terms grouping Gene Ontology terms obtained for DE miRNAs (Continued)

| **Gene Ontology Term** | **Global term** | **Month** |
| --- | --- | --- |
| positive regulation of cardiac muscle cell differentiation | Other | Upregulated at Month 6 |
| aorta smooth muscle tissue morphogenesis |  |  |
| cell migration involved in coronary vasculogenesis |  |  |
| positive regulation of myotube differentiation |  |  |
| negative regulation of calcium ion export across plasma membrane |  |  |
| positive regulation of cardiac muscle cell proliferation |  |  |
| positive regulation of heart rate |  |  |
| negative regulation of cardiac conduction |  |  |
| positive regulation of protein phosphorylation |  |  |
| negative regulation of cardiac muscle cell proliferation |  |  |
| negative regulation of cardiac muscle hypertrophy |  |  |
| positive regulation of voltage-gated potassium channel activity involved in ventricular cardiac muscle cell action potential repolarization |  |  |
| negative regulation of cardiac muscle myoblast proliferation |  |  |
| negative regulation of cell migration |  |  |
| negative regulation of cell population proliferation |  |  |
| negative regulation of xenobiotic detoxification by transmembrane export across the plasma membrane |  |  |
| negative regulation of delayed rectifier potassium channel activity |  |  |
| positive regulation of apoptotic process |  |  |
| activation of protein kinase B activity |  |  |
| positive regulation of calcium ion transmembrane transport via high voltage-gated calcium channel |  |  |
| negative regulation of glucose import |  |  |
| positive regulation of cell fate commitment |  |  |
| positive regulation of mesoderm formation |  |  |

**Table 9.** Global terms grouping Gene Ontology terms obtained for DE miRNAs (Continued)

| **Gene Ontology Term** | **Global term** | **Month** |
| --- | --- | --- |
| positive regulation of protein kinase B signaling | Other | Upregulated at Month 6 |
| positive regulation of pulmonary blood vessel remodeling |  |  |
| negative regulation of myoblast proliferation |  |  |
| regulation of release of sequestered calcium ion into cytosol by sarcoplasmic reticulum |  |  |
| negative regulation of prostaglandin biosynthetic process |  |  |
| ventricular septum morphogenesis |  |  |
| negative regulation of angiogenesis | Sprouting and angiogenesis |  |
| positive regulation of angiogenesis |  |  |
| positive regulation of sprouting angiogenesis |  |  |
| negative regulation of vascular associated smooth muscle cell proliferation | VSMCs proliferation/migration |  |
| positive regulation of cardiac vascular smooth muscle cell differentiation |  |  |
| regulation of phenotypic switching |  |  |
| establishment or maintenance of cell type involved in phenotypic switching |  |  |
| positive regulation of vascular associated smooth muscle cell apoptotic process |  |  |
| positive regulation of skeletal muscle cell differentiation |  |  |
| positive regulation of vascular associated smooth muscle cell migration |  |  |
| negative regulation of smooth muscle cell proliferation |  |  |

**Table 9.** Global terms grouping Gene Ontology terms obtained for DE miRNAs (Continued)

| **Gene Ontology Term** | **Global term** | **Month** |
| --- | --- | --- |
| negative regulation of blood vessel endothelial cell migration | Endothelial cell proliferation/migration | Downregulated at Month 6 |
| negative regulation of endothelial cell apoptotic process |  |  |
| negative regulation of blood vessel endothelial cell proliferation involved in sprouting angiogenesis |  |  |
| negative regulation of vascular endothelial cell proliferation |  |  |
| positive regulation of blood vessel endothelial cell migration |  |  |
| positive regulation of blood vessel endothelial cell proliferation involved in sprouting angiogenesis |  |  |
| positive regulation of ERK1 and ERK2 cascade | Inflammatory processes |  |
| negative regulation of tumor necrosis factor-mediated signaling pathway |  |  |
| positive regulation of MAPK cascade |  |  |
| negative regulation of cell adhesion |  |  |
| positive regulation of apoptotic signaling pathway |  |  |
| negative regulation of fibroblast apoptotic process |  |  |
| hypoxia-inducible factor-1alpha signaling pathway |  |  |
| positive regulation of inflammatory response |  |  |
| negative regulation of interferon-gamma production |  |  |
| negative regulation of macrophage migration |  |  |
| negative regulation of metalloendopeptidase activity |  |  |
| negative regulation of NF-kappaB transcription factor activity |  |  |
| negative regulation of apoptotic signaling pathway |  |  |
| negative regulation of nitric-oxide synthase activity |  |  |
| positive regulation of endothelial cell apoptotic process |  |  |
| positive regulation of phosphatidylinositol 3-kinase signaling |  |  |
| positive regulation of reactive oxygen species biosynthetic process |  |  |

**Table 9.** Global terms grouping Gene Ontology terms obtained for DE miRNAs (Continued)

| **Gene Ontology Term** | **Global term** | **Month** |
| --- | --- | --- |
| negative regulation of response to cytokine stimulus | Inflammatory processes | Downregulated at Month 6 |
| negative regulation of inflammatory response |  |  |
| negative regulation of cell migration | Other |  |
| positive regulation of protein kinase B signaling |  |  |
| negative regulation of cardiac muscle cell apoptotic process |  |  |
| plasma membrane raft assembly |  |  |
| negative regulation of sodium ion import across plasma membrane |  |  |
| negative regulation of cell population proliferation |  |  |
| negative regulation of amyloid-beta formation |  |  |
| negative regulation of aconitate hydratase activity |  |  |
| negative regulation of mitochondrial electron transport, NADH to ubiquinone |  |  |
| negative regulation of voltage-gated sodium channel activity |  |  |
| negative regulation of neuron projection development |  |  |
| positive regulation of cell migration |  |  |
| negative regulation of osteoblast differentiation |  |  |
| positive regulation of glucose catabolic process to lactate via pyruvate |  |  |
| positive regulation of iron ion import across plasma membrane |  |  |
| positive regulation of osteoblast differentiation |  |  |
| regulation of cellular response to hypoxia |  |  |
| cell growth involved in cardiac muscle cell development |  |  |
| negative regulation of osteoblast proliferation |  |  |
| negative regulation of protein K63-linked ubiquitination |  |  |
| positive regulation of vasculature development |  |  |
| negative regulation of protein serine/threonine kinase activity |  |  |
| tube formation |  |  |
| negative regulation of proteolysis |  |  |

**Table 9.** Global terms grouping Gene Ontology terms obtained for DE miRNAs (Continued)

| **Gene Ontology Term** | **Global term** | **Month** |
| --- | --- | --- |
| positive regulation of angiogenesis | Sprouting and angiogenesis | Downregulated at Month 6 |
| positive regulation of cell migration involved in sprouting angiogenesis |  |  |
| negative regulation of sprouting angiogenesis |  |  |
| negative regulation of angiogenesis |  |  |
| positive regulation of sprouting angiogenesis |  |  |
| negative regulation of vascular associated smooth muscle cell apoptotic process | VSMCs proliferation/migration |  |
| positive regulation of vascular associated smooth muscle cell apoptotic process |  |  |

**Table 9.** Global terms grouping Gene Ontology terms obtained for DE miRNAs (Continued)

| **Gene Ontology Term** | **Global term** | **Month** |
| --- | --- | --- |
| regulation of interleukin-10 production | Interleukin production | Upregulated at Month 9 |
| regulation of cell migration | Other |  |
| regulation of cell population proliferation |  |  |
| regulation of blood vessel endothelial cell proliferation involved in sprouting angiogenesis | Endothelial cells proliferation/migration | Downregulated at Month 9 |
| regulation of blood vessel endothelial cell migration |  |  |
| regulation of vascular endothelial cell proliferation |  |  |
| regulation of endothelial cell apoptotic process |  |  |
| regulation of inflammatory response | Inflammatory processes |  |
| regulation of MAPK cascade |  |  |
| regulation of ERK1 and ERK2 cascade |  |  |
| regulation of protein kinase B signaling | Other |  |
| regulation of cell migration |  |  |
| regulation of phosphatidylinositol 3-kinase signaling |  |  |
| regulation of proteolysis |  |  |
| regulation of cardiac muscle hypertrophy in response to stress |  |  |
| regulation of connective tissue replacement |  |  |
| regulation of vasculature development |  |  |
| regulation of sprouting angiogenesis | Sprouting and angiogenesis |  |
| regulation of angiogenesis |  |  |
| regulation of cell migration involved in sprouting angiogenesis |  |  |
